# Estradiol induces BDNF/TrkB signaling in triple-negative breast cancer to promote brain metastases

**DOI:** 10.1038/s41388-019-0756-z

**Published:** 2019-02-22

**Authors:** Maria J. Contreras-Zárate, Nicole L. Day, D. Ryan Ormond, Virginia F. Borges, Stuart Tobet, Brunilde Gril, Patricia S. Steeg, Diana M. Cittelly

**Affiliations:** 1 Department of Pathology, University of Colorado Denver, Anschutz Medical Campus, Aurora, CO USA; 2 Department of Neurosurgery, University of Colorado Denver, Anschutz Medical Campus, Aurora, CO USA; 3 Department of Medicine, Division of Medical Oncology, University of Colorado Denver, Anschutz Medical Campus, Aurora, CO USA; 4 Department of Biomedical Sciences, Colorado State University, Fort Collins, CO USA; 5 Women’s Malignancies Branch, National Cancer Institute, Bethesda, MD USA

**Keywords:** Breast cancer, Cancer microenvironment

## Abstract

Breast cancer brain metastases (BM) affect younger women disproportionally, including those lacking estrogen receptor (ER), progesterone receptor, and HER2 (known as triple-negative breast cancer; TNBC). Previous studies in preclinical models showed that pre-menopausal levels of estradiol (E2) promote TNBC-BM through incompletely understood mechanisms involving reactive astrocytes. Herein, a novel mechanism involving E2-dependent upregulation of brain-derived neurotrophic factor (BDNF) in astrocytes, and subsequent activation of tumor cell tropomyosin kinase receptor B (TrkB), is identified. E2 increased experimental BM of TNBC 4T1BR5 and E0771 cells by 21 and 3.6 fold, respectively, compared to E2-depleted mice. ERα^+^ reactive astrocytes were found at early and late stages of BM, and E2 upregulated BDNF in ER^+^ reactive astrocytes in vitro and in vivo. TrkB was expressed in TNBC brain-trophic cell lines, BM-patient-derived xenografts, and breast cancer BM. Conditioned media from E2-treated astrocytes (CM-E2) activated TrkB and downstream AKT, ERK, and PLC-γ signaling in TNBC cells, increasing their invasiveness and tumor-initiating capability in vitro. The promotion of BM by E2-activated astrocytes was found to be more complex, involving feedback loops and other receptor tyrosine kinases. In 4T1BR5 cells, there was a positive feedback loop whereby astrocytic BDNF induced cancer cell BDNF translation. Upregulation of cancer cell BDNF was required to promote full invasiveness of 4T1BR5 in response to CM-E2, and was observed in brain metastatic cells in E2-treated mice in vivo. Moreover, the non-competitive BDNF/TrkB inhibitor ANA-12 reduced E2-induced 4T1BR5 BM to levels similar to OVX mice. BDNF also activated EGFR in TrkB^+^EGFR^+^ TNBC cells, suggesting that E2 action through astrocytes activates redundant pathways promoting BM. These findings have important therapeutic implications, as they provide a rationale to use E2-depletion therapies or TrkB inhibitors to prevent or delay development of BM in younger women.

## Introduction

Brain metastases (BM) are a devastating complication of cancer in terms of cognitive, physical, and quality of life adverse outcomes (rev in [1, 2]). Among the common subtypes of breast cancer, BM preferentially develop in metastatic patients with HER2 -positive disease and triple-negative breast cancers (TNBC), which lack estrogen receptor (ER), progesterone receptor (PR), and HER2 alterations [3, 4]. Development of BM predicts poorest survival in TNBC patients and results in death within months of diagnosis [3, 5, 6]. While the increased metastatic potential and subsequent worse prognosis of TNBC has been attributed to the intrinsic genetics of these tumors, a young age at diagnosis predicts worse prognosis for various tumor subtypes, including TNBC and the luminal B subtype [7–11]. Young pre-menopausal women with TNBC showed increased incidence of BM (53%) compared to post-menopausal women (28%) [12]. These data suggested the novel hypothesis that pre-menopausal hormones such as estradiol (E2) may promote BM of TNBC by exerting effects on the brain microenvironment.

E2 plays pleiotropic roles in brain function through activation of ERs expressed in glial and neuronal cells [13–15]. Prior studies showed that reactive astrocytes, which surround and infiltrate human and experimental BM, expressed both ER genes (ERα and ERβ) and upregulated epidermal growth factor receptor (EGFR) ligands in response to E2, promoting migration, invasion, and proliferation of EGFR^+^ brain-tropic 231BR TNBC cells [16]. However, 40% of TNBC do not overexpress EGFR [17, 18], raising the possibility that alternative mechanisms contribute to E2 promotion of BM in TNBC.

Here we demonstrate that E2 promotes TNBC metastases by a novel mechanism involving E2-dependent paracrine and autocrine upregulation of BDNF, and subsequent activation of its receptor TrkB, expressed by cancer cells. TrkB has been shown to mark a subpopulation of putative cancer stem cells in recurrent TNBC, promote anoikis-resistance, and increase metastatic potential [19–23]. We show that E2-upregulated BDNF in ER^+^ astrocytes and cancer cells activated TrkB signaling and increased the invasiveness and tumor-initiating capacity of TNBC cells in vitro*.* Moreover, BDNF cross-activated TrkB and EGFR in cancer cells expressing both receptors, suggesting a novel cooperative interaction between these signaling pathways in TNBC. These studies provide a novel mechanism whereby E2 action in the brain microenvironment activates oncogenic signals in TNBC promoting BM, and provide a rationale for clinical testing of E2-depletion therapies and TrkB inhibitors in preventing development of BM.

## Results

### E2 promotes BM of EGFR^-^ TN cancer cells

**Fig. 1:**
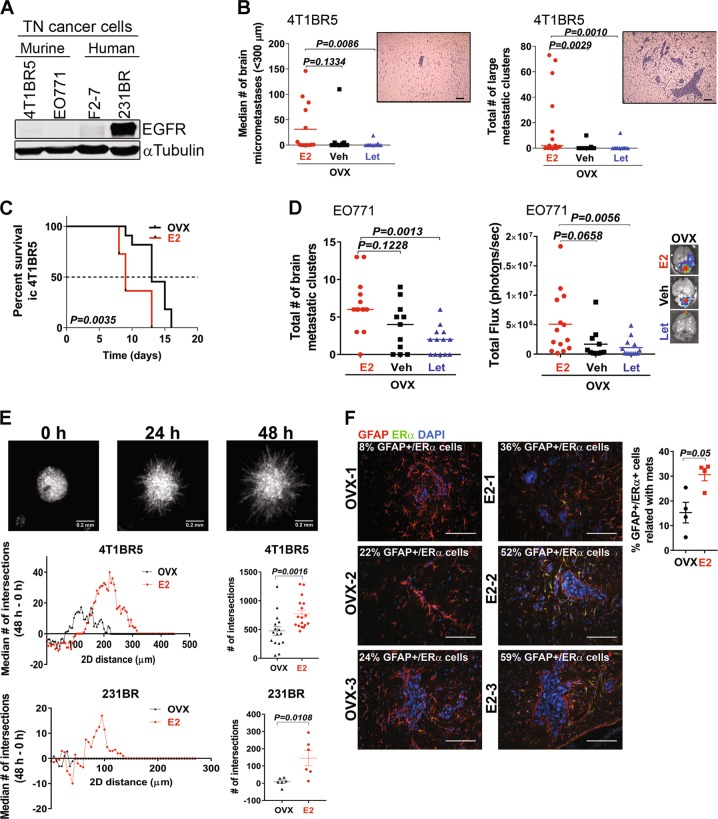
E2 promotes metastases of TN EGFR¯ 4T1BR5 cells. **a** EGFR expression in brain-metastatic TNBC cells (α-tubulin was used as loading control). **b** OVX BALB/c female mice supplemented with E2 (*n* = 14), placebo pellets (OVX, *n* = 15), or placebo pellets + letrozole (OVX + Letrozole, *n* = 15) were injected IC with 4T1BR5 cells. Left*:* Each dot represents the median number of micrometastases (<300 µm) per mouse and the line designates the group median. Representative H&E of a micrometastasis is shown. Right: Total number of metastatic clusters per mouse in the same experiment. Representative H&E stain of a metastatic cluster is shown. The data were analyzed using Kruskal–Wallis ANOVA followed by Dunn’s multiple comparisons test. **c** 4T1BR5 cells were injected IC in OVX female BALB/c mice supplemented with placebo or E2 pellets as in b (*n* = 11/group) and survival to humane end point was recorded. Log rank Mantel-Cox test was used to compare survival curves. **d** E0771-GFP-luc cells were IC injected in C57Bl/6 female mice treated as in b, (E2, *n* = 14; OVX, *n* = 11; OVX + let, *n* = 13). Left*:* Total number of brain metastatic clusters per mouse quantified as in b*.* Right*:* Metastatic burden quantified as total flux (photons/sec) in excised brains with representative images of luminescence signal in brains from each group (line marks group median). The data were analyzed using Kruskal–Wallis ANOVA followed by Dunn’s multiple comparisons test. **e** Invasion of 4T1BR5 and 231BR cells on organotypic brain slices from E2 or OVX BALB/c female mice was quantified as the number of intersections between cancer cells and concentric circles drawn from the edge of the sphere (Sholl analysis, see supplementary Fig 2 for details). Top shows representative image of a sphere at 0, 24, and 48 h after plating. Left, plots show the median number of new intersections/sphere in 5 µm increments from initial sphere edge 48 h after plating. Right graphs show total number of new intersections per sphere, 48 h after plating. *n* = 16 spheres/group for 4T1BR5 and *n* = 6/group for 231BR. Two-tailed Mann–Whitney test. Line represents median. **f** Double IF staining of ERα (green) and reactive astrocytes (GFAP, red) associated with BM in brain sections of OVX (*n* = 4) or E2-treated mice (*n* = 4). Left: representative image shows percentage of GFAP^+^ /ERα^+^ cells surrounding BM in OVX (*n* = 3) and E2-treated (*n* = 3) mice. Scale bar is 100 µm. Right, dots show the mean percentage of GFAP^+^/ERα^+^cells in 15 × 0.134 mm^2^ fields per mouse brain, two-tailed Mann–Whitney test. Line shows the group median

Prior studies showed that E2 promotes brain metastatic colonization of a brain-tropic subline of human TN MDA-MB-231 breast cancer cells (231BR) [24] via paracrine activation of EGFR [16]. These cells express higher levels of EGFR compared to other TNBC cell lines (4T1BR5, E0771, F2-7, Fig. 1a); thus, we assessed whether E2 could promote BM in the absence of EGFR overexpression. For this, a non-overexpressing (EGFR¯) subline of murine TN 4T1 cells (4T1BR5) and a TN cell line syngeneic to C57Bl/6 mice (E0771) were used. Ovariectomized (OVX) female mice were divided into three groups: implantation with slow release pellets containing (i) E2 (1 mg; maintains E2 levels equivalent to those found in pre-menopausal women; Supplementary Fig. 1a), (ii) placebo (OVX), or (iii) placebo mice additionally treated with aromatase-inhibitor letrozole (OVX + Letrozole) to block peripheral E2-synthesis. Two days post endocrine initiation, 4T1BR5 or E0771-GFP-luc cells were introduced via intracardiac (ic) injection and mice were euthanized 15 days later. BM were quantified histologically as previously described [25], or *ex-vivo* imaging of brains at euthanasia (for E0771-GFP-luc) was performed. E2-treated mice injected with 4T1BR5 cells showed a median of 31.32 ± 12.8 micrometastases per mouse, compared to 8.26 ± 7.3 and 1.46 ± 1.26 in OVX mice alone or OVX + letrozole treated mice, respectively, (*P* = 0.0086, a 21-fold E2 vs OVX + Let, Fig. 1b). Similarly, E2-treated mice showed an average of 18.5 metastatic clusters/mouse, while mice with ovarian estrogen depletion alone (OVX) or in combination with letrozole showed an average of 0.7 and 0.8 metastatic clusters/mouse (*P* = 0.0029 and *P* = 0.0010, respectively, Fig. 1b). Consistent with decreased BM, OVX mice showed improved overall survival compared to E2-treated mice injected IC with 4T1BR5 cells (Fig. 1c). Similar pro-metastatic effects of E2 were found in C57Bl/6 mice injected with E0771-GFP-luc cells using histological quantification (6.6 ± 3.5 vs. 1.8 ± 1.8 average metastatic clusters/mouse in E2 vs. OVX + let treated mice, respectively; 3.6 fold increase, *P* = 0.0013) and bioluminescence as endpoints (Fig. 1d, Supplementary Fig. 1b). Thus, E2 promoted BM and poorer survival in EGFR¯ TN cells, suggesting that mechanisms additional to EGFR activation promote TN-BM in response to E2.

To assess whether local mechanisms in the brain microenvironment mediated the pro-metastatic effects of E2, coronal brain sections of OVX and E2-treated mice were used as the substrate for invasion of 4T1BR5 and 231BR cells. Cancer cells seeded on top of brain slices from E2-treated mice showed increased number of invading branches (measured using Sholl analysis) [26] compared to cancer cells seeded on top of slices from OVX-treated mice (Fig. 1e, Supplementary Fig. 2). As these TNBC cells lack ERα and are unresponsive to direct effects of E2 [16] (Supplementary Fig. 3), these data suggest that E2-responsive cells within the brain niche promote metastatic traits in TNBC cells.

We reported that ER^+^ astrocytes surround experimental 231BR and human BM [16]. Double immunofluorescence (IF) demonstrated that astrocytes surrounding 4T1BR5 BM expressed membrane and nuclear ERα (Fig. 1f). ERα^+^ astrocytes were detectable in brains of mice carrying large mammary fat pad E0771 tumors without overt BM (Supplementary Fig. 4a), suggesting ER^+^ astrocytes can mediate pro-metastatic effects of E2 beginning in the early stages of BM. Moreover, an increased percentage of ERα^+^ astrocytes were found to be associated with late metastasis in E2-treated mice compared to OVX mice (30.6 ± 5.04% vs. 5.2 ± 8.3 GFAP^+^ERα^+^, respectively, *P* = 0.05, Fig. 1f, Supplementary Fig. 4b). Thus, ERα^+^ astrocytes can mediate the pro-metastatic effects of E2 throughout brain metastatic progression in EGFR¯ TN cells.

### E2 upregulates BDNF in ER^+^ reactive astrocytes in the brain microenvironment

**Fig. 2:**
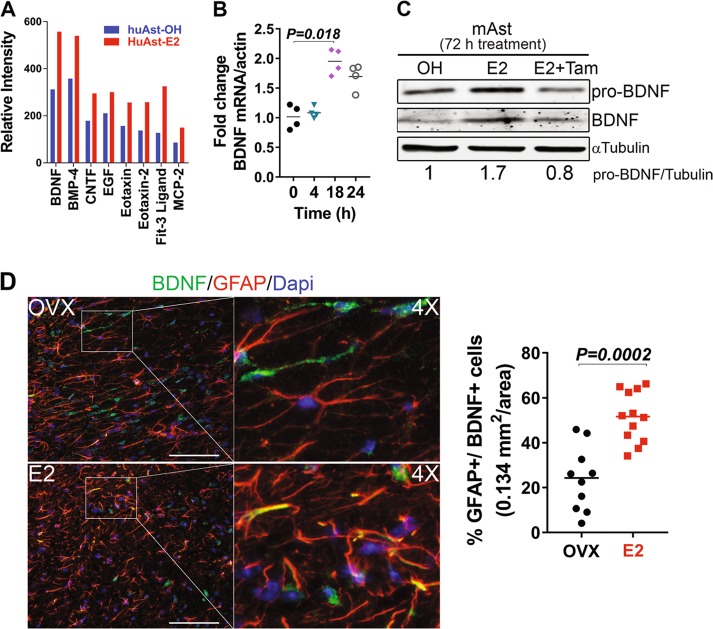
E2 upregulates BDNF in reactive astrocytes in vitro and in vivo. **a** Human primary astrocytes were treated with vehicle ethanol (OH) (Hu-Ast-OH) or 10 nM E2 (Hu-Ast-E2) for 24 h and cell lysates were assessed using growth factor arrays. Graph shows mean relative intensity of growth factors and cytokines with >1.4-fold increase in E2 compared to OH-treated astrocytes (*n* = 2 samples in independent arrays). **b** Primary mouse astrocytes were treated with vehicle (OH) or 10 nM E2 for the indicated times and BDNF mRNA levels were measured using qRT-PCR. Dots represent fold change mRNA levels normalized to β-Actin mRNA and relative to OH-treated. Line shows mean. The data were analyzed using Kruskal–Wallis test followed by Dunn’s multiple comparisons test. Adjusted P values are shown (*n* = 4). **c** WB shows pro-BDNF (34 kD) levels in mouse astrocytes (mAst) treated with vehicle (OH), 10 nM E2 alone or in combination with 1 µM Tam for 72 h. Numbers indicate ratio of BDNF/α-tubulin relative to OH-control (*n* = 2). **d** Double IF staining of BDNF (green) and reactive astrocytes (GFAP, red) in brain sections of OVX or E2-treated mice injected with 4T1BR5 cells. Left: representative image of GFAP^+^/BDNF^+^ cells in brains of OVX and E2-treated mice. The box indicates a region of interest shown at 4X magnification. Scale bar is 100 µm. Right: Dots show the mean percentage of GFAP^+^ cells expressing BDNF in 9 × 0.134 mm^2^ fields per mouse in OVX (*n* = 10) and E2-treated mice (*n* = 12). Two-tailed Mann–Whitney test was used for analysis. Line shows the group mean

A cytokine analyses of downstream effectors of E2 signaling in astrocytes was performed using lysates from human primary astrocytes treated with vehicle (huAst-OH) or 10 nM E2 (huAst-E2) for 24 h. Eight proteins (including EGF) were upregulated >1.4 fold in E2-treated astrocytes compared to OH-treated astrocytes. Among them, BDNF showed a 1.8-fold increase in E2-treated compared to OH-treated astrocytes, and was the growth factor with the highest relative expression (Fig. 2a). BDNF mRNA levels in astrocytes treated with E2 increased by 1.9 ± 0.2-fold at 18 h, compared to OH-treated astrocytes (*P* = 0.018 Fig. 2b). BDNF is translated as a precursor (pro-BDNF) that is cleaved into mature BDNF (BDNF). WB analysis showed that BDNF and pro-BDNF were expressed in vehicle-treated mouse primary astrocytes and upregulated by E2 (1.7 fold compared to vehicle, Fig. 2c). The ER selective agonist/antagonist tamoxifen (Tam) blocked E2-induced BDNF upregulation, suggesting this effect is dependent on astrocytic ERs (Fig. 2c, Supplementary Fig. 5a). To assess whether E2-induced upregulation of BDNF occurred in vivo, the number of reactive astrocytes (GFAP^+^) expressing BDNF was measured in brain sections from OVX and E2-treated BALB/c mice carrying 4T1BR5 metastases. The percentage of GFAP^+^ reactive astrocytes expressing BDNF (GFAP^+^BDNF^+^) was significantly increased in brains of E2-treated mice (51.4 ± 11.2% of astrocytes per 0.134 mm^2^) compared to OVX mice (23.8 ± 14.3%, *P* = 0.0002) (Fig. 2d, Supplementary Fig. 5b). These data suggest that E2 upregulates BDNF in astrocytes, altering BDNF/TrkB dependent signaling in the brain niche.

### BDNF receptor TrkB is expressed in a subpopulation of TNBC-BM

**Fig. 3:**
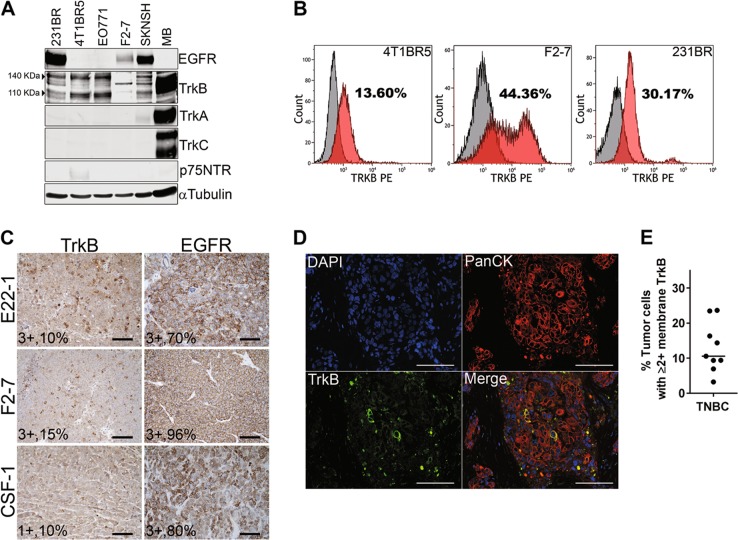
A subpopulation of cells within TNBC cell lines, PDXs, and clinical BM express TrkB. **a** WB shows EGFR, TrkB (140 and 110 kD isoforms), TrkC, p75NTR expression in murine (4T1BR5 and E0771) and human TNBC brain homing cells (231BR, F2-7). SKNSH and mouse brain (MB) were used as positive controls (*n* = 2). **b** Flow cytometry analysis shows the percentage of live cells expressing TrkB in 4T1BR5, F2-7 and 231BR cells. **c** Immunohistochemistry shows TrkB and EGFR expression in three TN BM-patient-derived xenografts. Numbers represent intensity and percentage of positive cells as scored by a pathologist. **d** TrkB (Green) in brain metastatic cells marked by pan cytokeratin (PanCK, red) assessed by double IF in a TNBM clinical sample. Scale bar is 100 µm. **e** Dots show % of tumor cells with membrane TrkB staining scored ≥ 2 + using Aperio (see supplementary Fig. 4 for detailed analysis method), in a cohort of breast cancer BM (*n* = 9)

We next assessed the expression of TrkB in TNBC cell lines with increased brain-metastatic potential: BM-patient-derived xenografts (BM-PDXs) and a panel of clinical TNBC-BM. Since there are a limited number of models of TNBC-BM, we derived a novel TNBC cell line (F2-7) from a BM-PDX [27]. Full length TrkB (possessing an intracellular kinase domain) in its glycosylated and unglycosylated forms (~140 and 110 kD) [28, 29] was detected in all brain-trophic cell lines from human (231BR, F2-7) and murine (4T1BR5, E0771) origin (Fig. 3a). BDNF binds with lower affinity to other receptors in the Trk family. Among these, TrkA and TrkC were not expressed in TN brain-trophic cell lines, and only 4T1BR5 cells showed low expression of p75^NTR^ (Fig. 3a). Flow cytometry analysis showed that these cell lines have heterogeneous expression of TrkB, ranging from 13.6 to 44.4% of TrkB^+^ cells (Fig. 3b). TrkB was restricted to a subpopulation of cells (10-15% of cells) in BM-PDXs and clinical samples (Fig. 3c, d). In a cohort of breast cancer BM, the extent of TrkB expression in the membrane of tumor cells was scored using Aperio Digital Imaging (Supplementary Fig. 6). Positive membranous TrkB staining (score ≥ 2 + ) was found in all TNBC (*n* = 9) specimens, with an average of 13.04 ± 7.1% tumor cells in TNBC BM (Fig. 3e). Thus, brain metastatic TNBC contains TrkB^+^ cells which can respond to E2-induced BDNF signaling in the brain microenvironment.

### E2-upregulated astrocytic-BDNF activates TrkB to promote invasion and mammosphere formation in TNBC cells in vitro

**Fig. 4:**
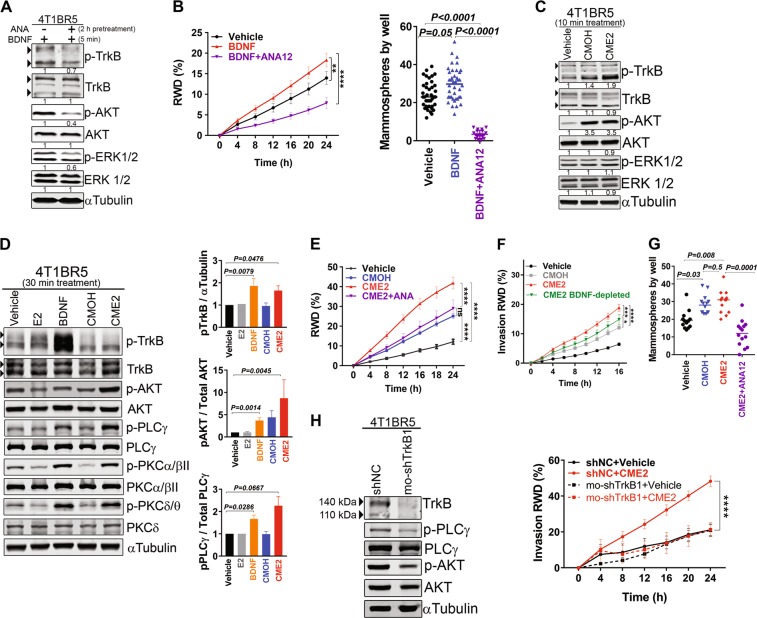
BDNF and CM-E2 activate TrkB and promote invasion and mammosphere formation in EGFR¯4T1BR5 cells. **a** 4T1BR5 cells were serum-starved overnight and pre-treated with vehicle or 10 µM ANA-12 2 h before treatment with 50 ng/ml BDNF for 5 min. Arrows indicate 140 and 110 kD TrkB isoforms. **b** Left*:* 4T1BR5 cells were plated in matrigel-coated plates, serum-starved for 16 h, and treated with vehicle or ANA-12 for 2 h. After creating the scratch wound, wells were coated with matrigel and vehicle or BDNF was added as a chemoattractant. Graphs show relative wound density (RWD) ± SEM (*n* = 6 wells/treatment). Right*:* 4T1BR5 cells were plated in Mammocult media at a density of 100 cells per well in a 96-well plate and treated with vehicle or 100 ng/ml BDNF alone or in combination with 1 µM ANA-12. Data show mammospheres at 10 days. **c** Signaling pathways activated in 4T1BR5 cells treated for 10 min with vehicle, CM-OH, or CM-E2. **d** Signaling pathways activated in serum-starved 4T1BR5 cells treated for 30 min with vehicle, 10 nM E2, 50 ng/ml BDNF, CM-OH, or CM-E2. Graphs shows fold change in levels of p-TrkB/tubulin, p-AKT/Total AKT, and p-PLCγ/Total PLCγ relative to vehicle-treated cells from at least 3 independent experiments. **e** 4T1BR5 cells were treated as in (**b**, left), and vehicle, CM-OH, or CM-E2 were used as chemoattractants in a scratch wound-modified invasion assay. **f** 4T1BR5 cells were treated as in (**e**), but CM-E2 was pre-incubated with a Trk-B Fc antibody (CM-E2- BDNF depleted) or an isotype IgG control (CM-E2). TrkB-Fc/BDNF or IgG complexes were depleted from CM-E2 using agarose beads. **g** 4T1BR5 cells were plated as in (**b**, right) and treated with vehicle, CM-OH, CM-E2 alone or in combination with 1 µm ANA-12. Graph shows number of mammospheres at day 10. **h**. Left: WB shows TrkB and downstream signaling in 4T1BR5 cells transduced with a shRNA lentiviral vector targeting mouse-TrkB (mo-shTrkB) or a non-targeting control (mo-shNC). Right*:* mo-shNC and mo-shTrkB cells were plated as in (**b**, left) and vehicle or CM-E2 were used as chemoattractants for the invasion assay. For WBs, numbers indicate ratio of protein/α-tubulin relative to control or time zero. For invasion assays, the data were analyzed using repeated measures ANOVA with post-hoc correction. Adjusted P values at 24 h are shown (*P < 0.05, **P < 0.01, ****P < 0.001). Graph represents one of at least two independent experiments. For mammosphere formation, graphs show # mammospheres/well (10 wells/treatment) in one of three independent experiments. The data were analyzed using Kurskal-wallis ANOVA with Dunn’s multiple comparison test

To determine whether paracrine BDNF stimulation could activate TrkB signaling in TNBC cells, serum-starved TNBC cells were treated with exogenous BDNF. Phosphorylation of TrkB (Y816) and activation of canonical BDNF/TrkB signaling mediators such as AKT, ERK, PLC-γ, PKC, and PKD were observed within 5 min to 1 h post-BDNF treatment (Supplementary Fig. 7a). In 4T1BR5 cells, BDNF-induced activation of TrkB and downstream signaling was blocked by a selective non-competitive antagonist of TrkB, ANA-12 (Fig. 4a). Activation of TrkB promotes migration of neurons and invasion of cancer cells [30, 31], thus we investigated if BDNF/TrkB activation promoted invasion in TNBC cells. Invasion was measured as the relative wound density (RWD) over time in a Matrigel-filled scratch wound assay. BDNF increased invasion of 4T1BR5 cells compared to vehicle-treated cells (13.0 ± 3.7 vs 18.3 ± 4.0% RWD, *P* = 0.0055) and ANA-12 abolished this effect (7.9 ± 3.1 RWD, *P* < 0.0001) (Fig. 4b). Similarly, BDNF increased migration of 231BR and E0771 cells compared to vehicle-treated cells (Supplementary Fig. 7b). To estimate whether BDNF affects the tumor-initiating ability of 4T1BR5 cells, a mammosphere formation assay was used. While BDNF did not influence proliferation of TNBC cells in 2D-culture (Supplementary Fig. 7c), BDNF induced a moderate increase in the number of mammospheres formed by 4T1BR5 cells compared to vehicle control (29.8 ± 8.5 vs 24.1 ± 7.0, respectively, *P* = 0.05). ANA-12 significantly decreased the number of mammospheres compared to both vehicle and BDNF-treated cells (3.4 ± 2.1, *P* < 0.0001) (Fig. 4b). This effect was not due to cytotoxicity, as ANA-12 did not alter cell confluency or viability over time (Supplementary Fig. 7d). Thus, BDNF/TrkB activation promotes metastatic traits in brain-trophic TNBC cells.

We next assessed whether E2-induced upregulation of BDNF in astrocytes activated TrkB and promoted invasion and mammosphere formation in 4T1BR5 cells. Conditioned media from E2-treated astrocytes (CM-E2) increased TrkB phosphorylation and downstream signaling compared to untreated cells or cells treated with conditioned media from vehicle-treated astrocytes (CM-OH) (Fig. 4c, d). E2 alone did not activate TrkB or downstream signaling in any of the TNBC cells tested (Fig. 4d, Supplementary Fig. 7e). While both CM-OH and CM-E2 activated AKT in 4T1BR5 cells at 10 min (Fig. 4c), only BDNF and CM-E2 induced activation of PLC-γ and PKC at later time points following stimulation (Fig. 4d). CM-E2 induced a larger increase in invasion of 4T1BR5 cells (41.9 ± 7.2% RWD) compared to CM-OH (25.1 ± 2.9% RWD *P* < 0.0001) or vehicle-treated cells (12.1 ± 3.0% RWD, *P* < 0.0001, Fig. 4e), and ANA-12 reduced invasion of CM-E2-treated cells to levels comparable to CM-OH-treated cells (29.0 ± 10.5% RWD, *P* < 0.0001, Fig. 4e). Depletion of BDNF by pre-treatment of CM-E2 with a TrkB-FC antibody reduced CM-E2-induced invasion (Fig. 4f), further supporting a role for astrocytic BDNF in promoting invasion in response to E2. In long-term mammosphere assays, CM-E2 increased mammosphere formation compared to vehicle-treated 4T1BR5 cells (30.0 ± 6.4 vs 19.5 ± 5.3, *P* = 0.008) and ANA-12 abolished this effect (12 ± 7.4, *P* < 0.0001) (Fig. 4g). Moreover, TrkB knockdown in 4T1BR5 cells did not alter their intrinsic invasive ability in organotypic brain slices (Supplementary Fig. 8a), but abolished CM-E2-induced invasion (Fig. 4h). Taken together, these data suggest that paracrine BDNF/TrkB activation in response to E2-stimulated astrocytes drives PLC-γ and PKC activation to promote invasion and mammosphere formation in 4T1BR5 cells.

### E2-stimulated astrocytes also promote BDNF upregulation in 4T1BR5 cells

**Fig. 5:**
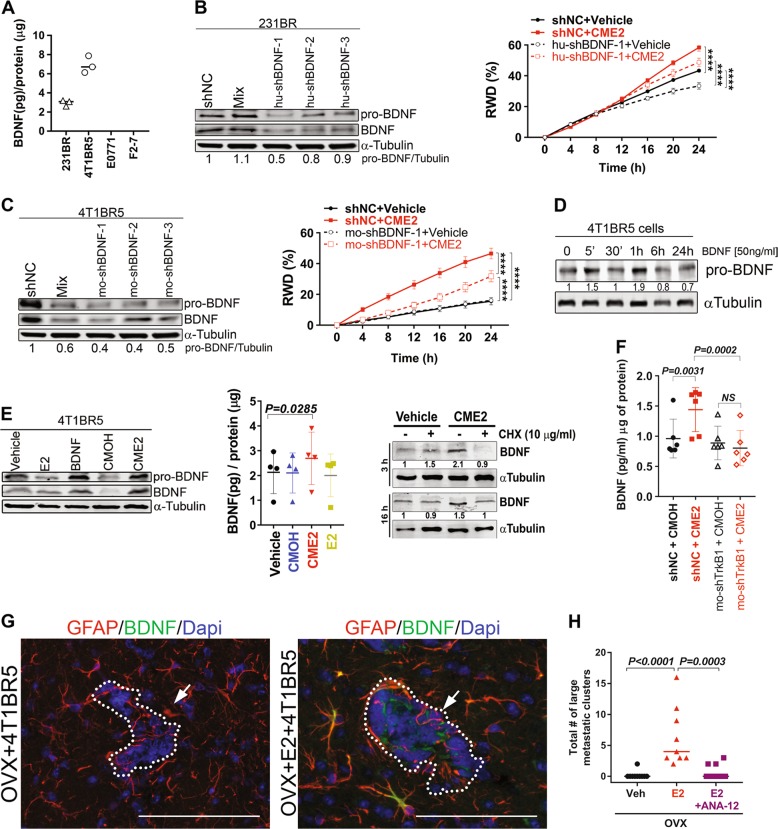
E2 signaling through astrocytes promotes cancer cell BDNF upregulation and blocking BDNF/TrkB signaling abolishes E2-induced BM. **a** BDNF expression in TNBC cells assessed by ELISA (*n* = 3). Cell lysates were obtained from cells cultured in 10% FBS-supplemented media. **b** Left: pro-BDNF and BDNF levels in 231BR cells expressing scramble control (shNC) or human BDNF targeting shRNAs. Right*:* Serum-starved shNC or hu-shBDNF-1 (1395) 231BR cells were treated with vehicle or CM-E2 as chemoattractants in scratch wound assays. Graphs show RWD ± SEM (4 wells/treatment). **c** Left*:* 4T1BR5 cells expressing sh NC or mouse BDNF targeting shRNAs. Right*:* Serum-starved shNC or mo-shBDNF-1-(5386) 4T1BR5 cells were treated with vehicle or CM-E2 as in **b** (6 wells/treatment). Data show one of 2 independent experiments; ****P < 0.0001 at 24 h using repeated measures ANOVA with Tukey’s multiple comparison test. **d** pro-BDNF and BDNF levels in 4T1BR5 cells treated with 50 ng/ml BDNF for indicated times. **e** Left*:* pro-BDNF and BDNF levels in 4T1BR5 cells treated for 24 h (WB) or 16 h (ELISA) with vehicle, 10 nM E2, 50 ng/ml BDNF, CM-OH, or CM-E2. Right*:* BDNF levels in 4T1BR5 cells treated with CM-E2 alone or in presence of 10 µg/ml Cycloheximide for 3 and 16 h (*n* = 3). **f** BDNF expression in shNC or shTrkB 4T1BR5 cells treated with CM-OH or CM-E2 for 16 h (*n* = 6) measured using ELISA. For experiments b-f, cells were cultured in hormone-deprived media (serum-free or 2% CS-FBS as indicated in Methods). **g** Double IF shows BDNF (green) expression in 4T1BR5 BM in brain sections of OVX or E2-treated mice injected with 4T1BR5 cells, 15 days after IC injection. Arrow indicates brain metastasis. Scale bar is 100 µm. For all WBs, numbers indicate ratio of protein/α-tubulin relative to control or time zero. For ELISAS, the data were analyzed using non-parametric ANOVA with Dunn’s multiple comparison test. **h**. 4T1BR5 cells were injected IC in OVX BALB/c female mice supplemented with E2 (*n* = 14) or placebo pellets (OVX, *n* = 15). E2-treated mice were administered daily IP injections of vehicle (E2) or ANA-12 (E2 + ANA12), and mice sacrificed 15 days later. Graph shows total number of brain metastatic clusters per mouse. The data were analyzed using Kruskal–Wallis ANOVA followed by Dunn’s multiple comparisons test

Autocrine BDNF/TrkB activation has been shown to promote metastatic traits in primary breast tumors. ANA-12 blocked invasion and mammosphere formation in 4T1BR5 cells below vehicle-treated levels (Fig. 4b–g) suggestive of autocrine BDNF/TrkB activation. 4T1BR5 and 231BR cells expressed BDNF (~ 7 pg/µg and 3 pg/µg protein, respectively), while F2-7 and E0771 cells lacked detectable BDNF expression in vitro (Fig. 5a). To assess the impact of autocrine and paracrine BDNF/TrkB activation in E2-induced invasion, three different shRNA lentiviral vectors were used to knockdown BDNF in 231BR and 4T1BR5 cells, and their ability to invade in response to vehicle or CM-E2 was assessed. One of three shRNAs decreased BDNF levels by 50 and 60% in 231BR and 4T1BR5 cells respectively, compared to cells expressing a non-targeting control (shNC) (Fig. 5b, c). 231BR shBDNF cells were less invasive than shNC cells treated with vehicle (33.4 ± 4.1 vs 43.3 ± 1.6 RWD, *P* < 0.001) or CM-E2 (48.8 ± 5.2 vs 58.4 ± 4.8 RWD, *P* < 0.001, Fig. 5b), but exhibited similar intrinsic ability to respond to CM-E2 (~30% increase in invasion compared to vehicle-treated cells). In contrast, shBDNF-4T1BR5 cells showed similar invasive ability compared to shNC-4T1BR5 cells treated with vehicle (15.6 ± 5.7 vs. 16.18 ± 1.1 RWD), but showed 30% less invasive ability when treated with CM-E2 compared to shNC cells (31 ± 8.9 vs 46.6 ± 8.4 RWD *P* < 0.0001, Fig. 5c). As these cells only differ in their ability to produce BDNF, this suggests that factors released by astrocytes in CM-E2 upregulate BDNF in cancer cells, promoting autocrine BDNF/TrkB activation.

In neurons, BDNF/TrkB activation upregulates autocrine BDNF secretion to induce a positive feedback loop, enhancing TrkB function. Consistently, exogenous BDNF induced upregulation of BDNF in 4T1BR5 cells within the first 24 h following stimulation (Fig. 5d). Western blot (WB) and ELISA analysis of 4T1BR5 cells stimulated with CM-E2 showed increased levels of BDNF compared to CM-OH or vehicle-treated cells and cycloheximide blocked this effect (Fig. 5e, Supplementary Fig. 8b), suggesting that CM-E2-dependent upregulation of BDNF depended on translational activation. TrkB knockdown in 4T1BR5 cells abolishes the ability of CM-E2 to increase BDNF levels (Fig. 5f), further suggesting the existence of a BDNF/TrkB positive feedback loop in these cells. Importantly, analysis of BDNF staining in 4T1BR5 experimental BM showed increased BDNF expression in cancer cells in E2-treated mice, compared with OVX-treated mice (Fig. 5g, Supplementary Fig. 9), suggesting that E2 promotes BDNF upregulation in BM.

To further assess how BDNF/TrkB activation (both autocrine and paracrine) could impact E2-induced BM, 4T1BR5 cells were injected IC in OVX mice treated with placebo (OVX), E2-pellets alone (E2), or in combination with ANA-12 (E2 + ANA-12). ANA-12 decreased E2-induced BM to levels similar to that of OVX-mice (6.3 ± 4.7 average metastatic clusters in E2-treated mice, vs 0.2 ± 0.2 in OVX vs 0.6 ± 1.1 in E2 + ANA-12 treated mice, *P* < 0.001, Fig. 5h). Thus, blockage of BDNF/TrkB abolishes the pro-metastatic effects of E2 in the brain in EGFR¯ TNBC cells.

### TrkB and EGFR signaling converge to promote invasion and mammosphere formation in EGFR^+^TrkB^+^ brain metastatic cells

**Fig. 6:**
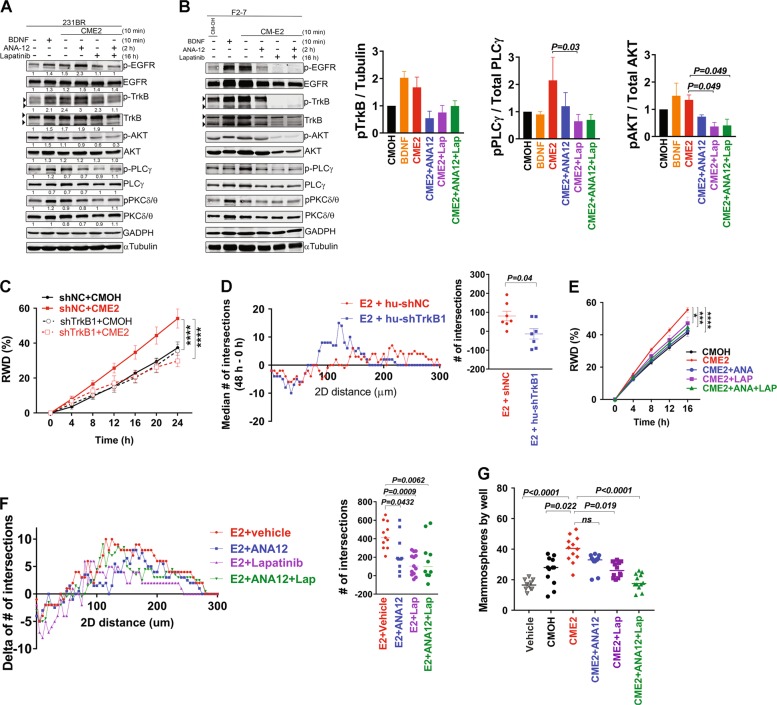
E2-induced activation of TrkB and EGFR signaling converge to promote invasion and mammosphere formation in TNBC. **a** 231BR and **b** F2-7 cells were starved in 5% charcoal-stripped FBS media for 12 h, treated with vehicle (DMSO), 1 µM Lapatinib for 8 h, 1 µM ANA-12 for 2 h, and then stimulated with vehicle, 100 ng/ml BDNF, CM-OH, or CM-E2 for 10 min. Alpha tubulin was used as loading control. Numbers indicate ratio of protein/α-tubulin relative to control. Graphs show fold changes of p-TrkB/Tubulin, p-PLCγ/total PLCγ and p-AKT/Total AKT relative to vehicle-treated cells from 2–4 independent experiments. **c** 231BR cells expressing shNC (shNC) or an shRNA targeting TrkB (hu-shTrkB1) were plated in matrigel-coated plates and serum-starved for 16 h. After scratch wound, invasion well were coated with matrigel and then CM-OH or CM-E2 used as chemoattractants. Graphs show average RWD ± SEM. The data were analyzed using repeated measures ANOVA with Tukey’s multiple comparison test. Adjusted P values *P < 0.05, **P < 0.01, ***P < 0.001 at 24 h. **d** shNC or hu-shTrkB 231BR cells were plated on organotypic brain slices from E2-treated mice and invasion assessed using Sholl analysis. Left: distribution of the median # of intersections away from initial sphere edge 48 h after seeding. Right*:* Total # of new intersections. **e** 231BR cells were plated in matrigel-coated plates, serum-starved for 16 h and pre-treated with vehicle, 1 µM Lapatinib, 1 µM ANA-12, or both. Invasion on matrigel-filled scratch wound was measured as in c. **f** 231BR cells were pretreated with ANA-12, Lapatinib or a combination, and plated on organotypic brain slices from E2-treated mice. Left: distribution of the median # of intersections away from initial sphere edge 48 h after seeding. Right*:* Total # of new intersections. Kruskal–Wallis with Dunn’s multiple comparisons test. **g** F2-7 cells were plated in EGF-free mammocult media (500 cells/well) and treated with vehicle, CM-OH, CM-E2 alone or in combination with 1 µM ANA-12, 1 µM Lapatinib, or both. Mammospheres were quantified at 14 days. Graph shows the number of mammospheres per well (*n* = 10 per treatment) in one representative experiment (from 2 repetitions). Data were analyzed using Kruskal–Wallis test with Dunn’s multiple comparison correction. For all graphs adjusted P values are shown. For all WBs α-tubulin was used as loading control and numbers indicate ratio of protein/α-tubulin relative to control or time zero

Given that E2-treated astrocytes activate both EGFR [16] and TrkB ligands on brain metastatic cancer cells and the majority of human BM are EGFR^+^, we assessed how EGFR and TrkB activation contributed to signaling and metastatic traits in EGFR^+^TrkB^+^ TNBC cells. The 231BR and F2-7 TNBC brain metastatic lines were used as both are EGFR^+^ and TrkB^+^ (Fig. 3a). Similar to 4T1BR5 cells, CM-E2 increased activation of AKT and PLC-γ as compared to vehicle (Fig. 6a, b Supplementary Fig. 10a,b). CM-E2 induced EGFR (Y1068) and TrkB activation in 231BR and F2-7 cells, compared to vehicle or CM-OH-treated cells (Fig. 6a, b). Intriguingly, BDNF activated both TrkB and EGFR in these cell lines (Fig. 6a, b), suggesting cross-activation of EGFR by BDNF. Consistent with this, ANA-12 decreased CM-E2-induced TrkB and EGFR signaling in F2-7 cells, and the EGFR/HER2 inhibitor Lapatinib decreased both EGFR and TrkB phosphorylation of CM-E2-treated F2-7 and 231BR cells. ShRNA-mediated downregulation of TrkB in 231BR cells (Fig. 6c, d, Supplementary Fig. 10), or treatment with ANA-12 and Lapatinib alone were sufficient to reduce invasion in response to CM-E2 in vitro and in E2-treated organotypic brain slices (Fig. 6e, f). In long-term mammosphere assays (14 days), ANA-12 was less effective than Lapatinib in decreasing CM-E2-induced mammosphere formation, but the combination of ANA-12 + Lapatinib showed a cumulative effect (Fig. 6g). Together, these data suggest that redundant activation of BDNF/TrkB and EGFR signaling contribute to mediate the pro-metastatic effects of E2 in EGFR^+^TRkB^+^ TNBC BM.

## Discussion

Using brain-tropic as well as non-selected models of TNBC, we have defined a novel mechanism by which E2-dependent upregulation of brain-derived neurotrophic factor (BDNF) in astrocytes, and subsequent activation of tumor cell tropomyosin kinase receptor B (TrkB) in TNBC cells, promotes brain metastatic colonization. Consistent with the known role of E2 in modulating neuroinflammatory responses in the brain via activation of ERs in glial cells [32, 33], ERα was expressed in reactive astrocytes at early and late stages of BMs in the 4T1BR5 model. Reactive astrocytes surround cancer cells early during brain colonization and are critical for the establishment and growth of BM [34–40], thus, it is likely that E2 action on ER^+^ astrocytes influences multiple steps in the brain colonization cascade. ERα^+^ reactive astrocytes associated with late BM increased in E2-treated mice compared to OVX-mice, suggesting the existence of a positive feedback loop between E2 and ERs in these cells. While quantifying the percentage of ER^+^ cells in the subset of GFAP^+^ cells limits the bias introduced by increased number of GFAP^+^ cells, upregulation of ERs in reactive astrocytes upon brain injury in primates has been reported [41], raising the possibility that increased ERα results from more BM-associated reactive astrocytes in E2-treated mice. Future studies will determine how astrocytic ERs are regulated in response to E2 and elucidate their role during brain metastatic progression.

BDNF is best known as a neurotrophic factor that promotes survival of neurons and plays critical roles during brain development [42, 43]. BDNF levels are very low (pg/g) in the unlesioned adult brain [44, 45], but injuries and various signals lead to upregulation and secretion of BDNF by astrocytes [46–48]. In this study, E2 upregulated transcription of BDNF leading to increased pro-BDNF and mature BDNF protein levels in astrocytes (Fig. 2a). Secreted pro-BDNF is cleaved to BDNF by the tissue plasminogen activator (tPA)/plasmin protease system in the brain [49–51], suggesting that astrocytic BDNF reaches cancer cells within the brain niche as mature BDNF. E2 has been shown to upregulate BDNF in some neuronal types [52, 53], thus, it is possible that neurons and other ER^+^ cells in the brain microenvironment could contribute to the pro-metastatic effects of E2 in the brain niche. Whereas BDNF^+^ neuronal bodies were observed in some brain areas in both OVX and E2-treated mice (Fig. 2d), neuronal BDNF expression was associated with specific brain-locations rather than with metastasis (*not shown*). Further studies using genetically engineered models to ablate ER in astrocytes are warranted to assess the extent to which astrocytic ERs and BDNF are required for E2-induced BMs, and how other ER^+^ cells in the brain niche contribute to the overall effects of E2 in promoting BM.

**Fig. 7:**
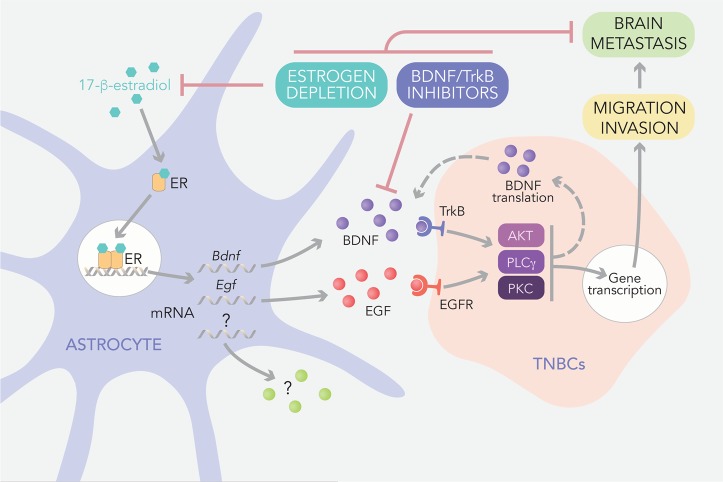
Current understanding of mechanisms underlying pro-metastatic effects of E2 in the brain. Reactive astrocytes express ERs and respond to pre-menopausal E2 levels by upregulating BDNF, EGF, and other growth factors and chemokines. BDNF can activate TrkB and/or EGFR in TNBC cells expressing these receptors, leading to activation of survival and invasive pathways. Paracrine action of E2 also leads to autocrine BDNF upregulation, supporting a positive feedback loop that enhances TrkB signaling in cancer cells. Full estrogen depletion and Trk inhibitors emerge as potential therapeutic alternatives in the prevention or treatment of TNBC BM

Surprisingly, E2 increased BDNF in astrocytes and by a paracrine mechanism, in cancer cells. A self-amplifying autocrine action of BDNF has been demonstrated in neurons, whereby extracellular BDNF triggers BDNF secretion from neurites and promotes surface insertion of TrkB receptor via Pi3K activation, amplifying BDNF/TrkB signaling [54]. Here, BDNF and E2-treated astrocytes triggered BDNF protein synthesis, and knockdown of TrkB in cancer cells abolished this upregulation, demonstrating the existence of a similar positive feedback loop of BDNF/TrkB in cancer cells. This novel finding demonstrates the complexity of the interactions between cancer cells and the brain niche and provides additional evidence that paracrine action of E2 alters critical pathways driving BM. While these studies cannot define the exact contribution of astrocytic vs cancer-cell BDNF to E2-induced BMs, targeting BDNF/TrkB function in vivo significantly blocked BM, providing compelling evidence that this BDNF/TrkB signaling is critical for E2-induced brain colonization. A model of the mechanisms by which E2 influence TNBC BM is depicted in Fig. 7.

BDNF-mediated activation of EGFR has been observed in lung cancer [31] and suggests BDNF/TrkB/EGFR cross-talk is a more general mechanism promoting BM. Recent studies reported a similar convergence between BDNF/TrkB and HER2 in BM [55], suggesting that E2-upregulated BDNF/TrkB activation may also promote BM in younger women with HER2^+^ tumors. Further studies are needed to define the therapeutic value of TrkB and EGFR inhibition for breast cancer BM.

Finally, these studies have broader therapeutic implications. BM are prevalent in metastatic TNBC and often occur in the setting of uncontrolled systemic disease. While we focused here on the brain-specific mechanisms underlying E2-fueling of BM, E2 promoted systemic metastasis in mice injected with E0771-GFP-luc cells. This is consistent with prior reports showing that E2 can promote tumor growth and metastasis via mobilization of myeloid-derived suppressor cells [56–58]. Thus, E2-depletion therapies may benefit overall survival in younger women with TNBC by decreasing both brain and systemic metastasis. The extent to which ovarian E2 depletion alone decreased metastasis varied among cell lines, as 4T1BR5 cells improved survival. Since OVX mice still died, it is possible that brain micrometastasis observed in OVX mice can progress in the presence of brain-synthesized estrogens [59–61]. Additionally, 4T1BR5 cells are not labeled, thus, it cannot be discounted that some extracranial metastasis affect survival. Given that full estrogen depletion (OVX + Letrozole) was more effective in decreasing BM in the E0771 model, blockage of ovarian and peripheral E2 (i.e., brain, adrenal, fat) may be necessary to prevent brain metastatic progression in TNBC. These findings also raise the possibility that peripheral imbalances in estrogen synthesis due to obesity, which disproportionately affects African American women with TNBC, could promote rapid progression of BM in this group. Taken together, these studies provide rationale to use aromatase inhibitors in the prevention of BM in younger women with TNBC, and for further pre-clinical testing of estrogen depletion therapies and Trk inhibitors as co-adjuvants for treatment of established metastases.

## Methods

### Human-derived BM

De-identified human samples were obtained from archival paraffin embedded tissue from consenting donors, under approved IRB protocols at the University of Colorado.

### Cell lines

Brain tropic (cells with increased brain-metastatic potential) derivatives of the TNBC MDA-MB-231 cells (231BR), TNBC BM-PDX (F2-7) cell line, and murine TN cell line 4T1 (4T1BR5, a gift from Dr. Suyun Huang) were maintained as described [24, 27, 62]. E0771 cells were purchased from CH3 BioSystems, LLC (Buffalo, NY) and maintained in RPMI supplemented with 5% FBS. E0771 cells expressing GFP-luciferase were used in vivo. Cell lines were free of mycoplasma (MycoAlert^TM^ PLUS-Lonza) and identity of human cells validated within 6 months of receipt by STR analysis (University of Colorado Tissue Culture Core). All experiments involving E2 were performed in serum-free media or supplemented with 2% CS-FBS.

### Primary astrocytes

Primary astrocytes were cultured as described [63]. To obtain 10× concentrated astrocytic-conditioned media (CM), 100% confluent astrocyte cultures were incubated for 72 h with serum-free phenol-red free media containing vehicle (CM-OH) or 10 nM E2 (CM-E2). Supernatants were concentrated 10× using a 3000 Da cutoff filter.

### qRT-PCR

Total RNA was isolated using Trizol and cDNA was synthesized using the Verso cDNA Synthesis Kit (Thermo Scientific). β-actin was used for normalization. Relative mRNA levels were calculated using the comparative Ct method (ΔΔCt).

### Migration and invasion assays

Cells were plated (40,000 cells/well) on a 96-well Essen ImageLock plate. Adherent cells were serum-starved for 16 h and a scratch wound was made using a 96-pin WoundMaker (Essen Bioscience), then treated as described. Wound images were taken every 4 h for 24 h and the relative wound confluency was calculated at each time point. When indicated, cells were pretreated with vehicle (DMSO), 1 µM Lapatinib for 12 h or 1 µM ANA-12 for 2 h prior to scratch wound. Treatments were performed in 5-replicates in at least two independent experiments. For invasion assays, cells were treated as above, but wounds were filled with matrigel.

### Invasion of organotypic brain slices

Coronal brain slices (400 µm) from OVX or E2-treated-female mice were plated on 12-well/transwells with 500 µl of 10% FBS phenol-red-free media. GFP^+^ cancer cells were grown as mammospheres for 10 days, resuspended in 3 µl of serum-free media and plated on top of brain slices (5–20 mammospheres/slice). GFP^+^ cells were imaged using fluorescent Stereo Microscope Leica M165FC at 0 h and 48 h after plating. Images were exported to ImageJ software (NIH) and spread of GFP^+^ cells (green mask) away from the edge of the mammosphere were quantified using Sholl analysis [26].

### Mammosphere formation assay

A total of 100–500 cells were suspended in 50 µl of mammocult media containing 1% methylcellulose, plated in ultra-low attachment 96-well plates and treated as indicated. Whole well images were taken 10 (231BR, 4T1BR5) or 14 (F2-7) days later using Incucyte zoom system. Mammospheres (Green mask) were analyzed using the command analyze particle in ImageJ software (NIH), parameters were area ≥ 0.005 mm^2^ and circularity 0.5–1.

### Reagents

A complete list of antibodies and key reagents used is in Supplementary Table 1.

### Experimental BM

Animal studies were approved by University of Colorado Institutional Animal Care and Use Committee. BM were developed by injecting 75,000 4T1BR5 or E0771-GFP-luciferase cells the left ventricle of ovariectomized 6–8-week-old female BALB-c or C57Bl6 mice (Jackson Labs). Mice were implanted with pellets containing either 17-β Estradiol (1 mg) or placebo (cellulose), two days before intracardiac inoculation. Used E2 dose is that required to promote tumor growth of ER^+^ dependent breast cancer cells in mice, and is within serum-levels in pre-menopausal women (~50–150 pg/ml, Supplementary Fig. 1a). Letrozole, an aromatase inhibitor known to cross the BBB [64], was diluted in 0.3% hydroxyl-propyl cellulose at 0.2 mg/ml and injected subcutaneously (10 µg/day). ANA-12 was diluted in peanut oil and administered IP at 0.5 mg/kg BW/daily. Mice were randomized to assign treatments; cell-injections and metastasis quantification were performed by investigators blind to the experimental groups. Sample size was calculated at 80% power, two sided tests and *α* = 0.05. Individuals were excluded from analysis if tumor cells grew around the heart (indicative of a failed ic injection) at euthanasia.

### Digital imaging

For IF analysis images were collected using a Nikon Eclipse Ti-S inverted microscope. ROIs were made using Confocal Uniovi 1–51 an ImageJ bundle [65]. Minor linear adjustments to brightness and contrast were performed identically and in parallel. For quantification, the total number of GFAP^+^DAPI^+^ and GFAP^+^BDNF^+^ or GFAP^+^ERα^+^ cells were counted using Nis-Elements software (version 4.30.01), and the percentage of GFAP^+^ cells expressing BDNF or ERα was calculated. WBs were imaged and quantified using Odyssey CLx Imaging System and Image Studio Software v.5.2.5 LI-COR Biosciences.

### Statistics

Statistics were done using Graphpad Prism 7.3 software. Two-tailed T test, one way ANOVA, or repeated measures ANOVA followed by multiple comparison post hoc tests were used as appropriate. When samples did not comply any normality assumption, non-parametric test (Two-tailed Mann–Whitney or Kruskal–Wallis test) were used. *P* < 0.05 was considered significant and test assumptions were checked for all analysis. Statistical analysis is reported with each figure legend. Adjusted *P* values are shown in all graphs.

## Supplementary information

Supplementary Table 1.

Supplementary Figure Legends.

Supplementary Figure 1.

Supplementary Figure 2.

Supplementary Figure 3.

Supplementary Figure 4.

Supplementary Figure 5.

Supplementary Figure 6.

Supplementary Figure 7.

Supplementary Figure 8.

Supplementary Figure 9.

Supplementary Figure 10.
