# Supplementary material for: Estradiol induces BDNF/TrkB signaling in triple-negative breast cancer to promote brain metastases
Source: Oncogene. 2019 Feb 22;38(24):4685–99. doi: 10.1038/s41388-019-0756-z (PMC6565485; doi:10.1038/s41388-019-0756-z)
Supplement: Supplementary file 1 — Supplementary Table 1. [file 41388_2019_756_MOESM1_ESM.docx]

**Supplementary Table 1. List of antibodies, shRNAs and reagents used.**

| **Target** | **Host** | **Company** | **Catalog number** | **Applications** |
| --- | --- | --- | --- | --- |
| **AKT** | Rabbit | Cell Signaling | cs9272S | WB |
| **BDNF**  **BDNF** | Rabbit  Rabbit | Santa Cruz  Abcam | sc20981  ab108319 | WB  WB |
| **EGFR** | Mouse | Sigma-Aldrich | E3138 | IF |
| **EGFR** | Rabbit | Cell Signaling | cs4267S | WB |
| **ERK1/2** | Rabbit | Cell Signaling | cs9102S | WB |
| **GFAP** | Rat | Invitrogen | 13-0300 | IF |
| **p75NGFR** | Rabbit | Abcam | ab52987 | WB |
| **pAKT (S473)** | Rabbit | Cell Signaling | cs4060S | WB |
| **pan-cytokeratin** | Mouse | Dakocytomation | M0821 | IF |
| **pEGFR (Y1068)** | Mouse | Cell Signaling | cs2236S | WB |
| **pERK1/2 (T202/T204)** | Rabbit | Cell Signaling | cs9101S | WB |
| **PKCα** | Rabbit | Cell Signaling | cs2056 | WB |
| **PKCδ** | Rabbit | Cell Signaling | cs2058 | WB |
| **PLCg1** | Rabbit | Santa Cruz | sc81 | WB |
| **pPKC (pan) (βIIS660)** | Rabbit | Cell Signaling | cs9371 | WB |
| **pPKCa (S657)** | Rabbit | Millipore | 06-822, | WB |
| **pPKCα/βII (T638/641)** | Rabbit | Cell Signaling | cs9375 | WB |
| **pPKCδ/θ (S643/676)** | Rabbit | Cell Signaling | cs9376 | WB |
| **pPKD/PKCμ (S744/748)** | Rabbit | Cell Signaling | cs2054 | WB |
| **pPLCg1 (Y783)** | Rabbit | Cell Signaling | cs14008 | WB |
| **pTrkA (Y490)/pTrkB(Y516)** | Rabbit | Cell Signaling | cs4619S | WB |
| **pTrkB (Y816)** | Rabbi | Millipore | ABN1381 | WB |
| **TrkA** | Rabbit | Abcam | ab76291 | WB |
| **TrkB** | Rabbit | Abcam | ab18987 | WB |
| **TrkB** | Rabbit | Cell Signaling | cs4607S | Flow, IF |
| **TrkC** | Rabbit | Cell Signaling | cs3376 | WB |
| **α-tubulin** | Mouse | Sigma-Aldrich | T5168 | WB |

**Secondary antibodies**

| **Target** | **Host** | **Conjugated** | **Company** | **Catalog number** | | **Applications** |
| --- | --- | --- | --- | --- | --- | --- |
| **Mouse** | Goat | Alexa Fluor 568 | Life technology | A11031 | IF | |
| **Mouse** | Goat | Alexa Fluor 680 | Life technology | A21058 | WB | |
| **Mouse** | Goat | IRDye 800CW | LI-COR Biosciences | 926-32280 | WB | |
| **Rabbit** | Goat | Alexa Fluor 488 | Life technology | A11034 | IF | |
| **Rabbit** | Goat | Alexa Fluor 680 | Life technology | A21109 | WB | |
| **Rabbit** | Goat | IRDye 800CW | LI-COR Biosciences | 926-32211 | WB | |
| **Rat** | Donkey | Alexa Fluor 594 | Life technology | A21209 | IF | |

**ShRNAs**

| **shRNAs** |  | **Target** | **Source** |
| --- | --- | --- | --- |
| hu-shBDNF1 | TRCN0000371395 | Human BDNF | Sigma Mission shRNA library |
| hu-shBDNF2 | TRCN0000058209 | Human BDNF | Sigma Mission shRNA library |
| hu-shBDNF3 | TRCN0000058208 | Human BDNF | Sigma Mission shRNA library |
| mo-shBDNF1 | TRCN0000065386 | Mouse BDNF | Sigma Mission shRNA library |
| mo-shBDNF2 | TRCN0000065387 | Mouse BDNF | Sigma Mission shRNA library |
| mo-shBDNF2 | TRCN0000065383 | Mouse BDNF | Sigma Mission shRNA library |
| hu-shTrkB1 | TRCN0000002242 | Human TrkB | Sigma Mission shRNA library |
| mo-shTrkB1 | TRCN0000023699 | Mouse TrkB | Sigma Mission shRNA library |
| mo-shTrkB2 | TRCN0000361390 | Mouse TrkB | Sigma Mission shRNA library |
| shNC | SHC216 | non-targeting control | Sigma Mission shRNA library |

| **Reagent** | **Company** | **Catalog number** |
| --- | --- | --- |
| Lapatinib | Selleckchem | S2111 |
| Letrozole | Selleckchem | S1235 |
| ANA-12 | Selleckchem | S7745 |
| 17-β-estradiol | Sigma | E2758 |
| 4-OH-Tamoxifen | Sigma | H7904 |
| BDNF | Peprotech | 450-02 |
