# Supplementary material for: Estradiol induces BDNF/TrkB signaling in triple-negative breast cancer to promote brain metastases
Source: Oncogene. 2019 Feb 22;38(24):4685–99. doi: 10.1038/s41388-019-0756-z (PMC6565485; doi:10.1038/s41388-019-0756-z)
Supplement: Supplementary file 2 — Supplementary Figure Legends. [file 41388_2019_756_MOESM2_ESM.docx]

**Sup Fig. 1: a** Levels of E2 in serum from E2, OVX and OVX+letrozole treated mice measured by ELISA. Samples were collected at euthanasia. **b** Systemic effects of E2 in metastatic colonization in mice injected with E0771-GFP-luc cell shown in Fig 1d. *Left:* Extra-cranial total flux from E2, OVX and OVX+letrozole-treated mice. *Right:* Representative images of mice showing extent of systemic metastatic burden per group.

**Sup Fig. 2:** Sholl analysis of invasion of cancer cells on organotypic brain slices**.** Images at 1.5X show multiple spheres in brain slice (**a**) were reimaged at 2.5X to map individual spheres (**b**), and at 12X to acquire high resolution images of single spheres (**c**) and 0 (**a-c**) and 48 h (**d-f**) after plating. **g** 12X images were adjusted for brightness and contrast and thresholded to represent original image using Fiji-ImageJ(63). **h** A mask representing GFP+ cells after thresholding was created for images at time 0 and the Sholl analysis plugin(26) was run. **i** In this analysis, the image center was marked, concentric circles at a chosen 5 µm radius step size were created, and the number of intersections at each distance were quantified. A closer view of marked points is shown in (**j**). The same analysis was performed in images of spheres 48 h later (**k-m**). **n** The number of new interceptions defined as those existing beyond the edge of the sphere at time 0h (distance marked by blue line), were used as a measure of invasion. Data in **Fig.** **1e** shows the number of new intersections at given distances from sphere’s edge at time zero (a measure of the distance traveled by invading cells), and the total number of new intersections per sphere (a measure of the overall branching of the cancer cells away from the sphere center).

**Sup Fig 3:** E2 does not alter migration**,** invasion or proliferation of TNBC cell lines. **a** 231BR, 4T1BR5 and E0771 were plated in 10% Charcoal stripped FBS (CSF)- phenol red free DMEM. After scratch wound, cells were treated with vehicle (ethanol) or 10nM E2 in 5% CSF-media. Graphs shows RWD +/- SEM. **b** Cells were plated as in a, but scratch wound was filled with matrigel. **c** Cells were plated at 5000 cells/well and cultured with 10% FBS- phenol red free DMEM supplemented with vehicle or 10 nM E2 for the indicated times. Graphs shows confluence (%) over time.

**Sup Fig 4:** **a** ERα expression in reactive astrocytes in brains from mice carrying E0771 tumors in the mammary fat pad, at early stages of metastatic dissemination. Left: representative image shows percentage of GFAP+/ERα+ cells in brains of OVX (n=4) and E2-treated (n=4) mice carrying ~2cm-diameter E0771-GFP-luc tumors in the mammary fat pad. Scale bar is 100 µm. *Right*, dots show the percentage of GFAP^+^/ERα^+^cells in at least 5 x 0.134 mm^2^ fields per mouse brain, two-tailed Mann-Whitney test. Line shows the group median. **b** Single channel images of Dapi, GFAP, and ERα corresponding to merged colored-image shown in Fig. 1f

**Sup Fig. 5 a.** Full image WB for Fig 2c using two BDNF antibodies*. Left:* H117 (SC 20891) recognizes mainly murine pro-BDNF (predicted MW, 35KDa). *Right:* Ab ERP1292 (Abcam 108319) recognizes multiple bands, including pro-BDNF (35KDa) and mature BDNF (15KD). Additional 28 kDa (possible multimer) and unspecific bands are detected by this antibody according to manufacturer validation. **b** Single channel images of GFAP, BDNF and DAPI corresponding to merged colored-image shown in Fig. 2d.

**Sup Fig. 6** Aperio software analysis for quantification of TrkB in clinical samples**.** Entire histological sections were imaged with Aperio ScanCope T3 scanner at 0.25 µm/pixel. Sample areas for quantification were annotated using Aperio analysis tools and a minimum of 4 mm^2^ tumor area (5000-20.000 tumor cells) were annotated from each sample. Stroma and necrotic areas were excluded from analysis. Algorithms developed in the Pathology Department at University of Colorado for HER2 or ER staining in clinical samples were used to assess membranous staining of TrkB. Clinical samples with scores of 2+ (moderate Intensity, orange masks) and 3+ (strong intensity, red masks) were considered positive.

**Sup Fig. 7** Exogenous BDNF activates TrkB, promotes invasion and does not affect proliferation of TNBC cells. **a** WB of serum-starved 4T1BR5, E0771 and F2-7 cells starved with 10% charcoal stripped fetal bovine serum (CSFBS) and treated with 50 ng/ml BDNF for the indicated times. Numbers indicate ratio of protein/α-tubulin relative to time zero. **b** E0771 and 231BR cells were serum-starved overnight and treated with 50 ng/ml BDNF as chemoattractant in scratch wound assays. Graphs shows RWD ± SEM. Data was analyzed using repeated measures one way ANOVA test. Adjusted P values *P<0.05, ***P<0.001 at 40 h to E0771 cells or 16 h to 231BR cells. **c** 4T1BR5, E0771 and 231BR cells (1000 cells/well) were plated in 96 well plate with 100 µl of media contained 2% FBS alone or with 50 ng/ml BDNF or 100 ng/ml BDNF and cell confluence measured using SRB assay. Bars represents folds change relative to day zero ± SEM. n=5 wells. Data show one of three independent experiments. **d** Proliferation assay of 4T1BR5, E0771 and 231BR cultured media containing 1%-CSFBS supplemented with vehicle or 1µM ANA-12 for up to 6 days. Graphs shows % confluency ± SEM. **e** BDNF signaling in 231BR, 4T1BR5 and F2-7 cells serum-starved overnight treated with 10 nM E2, vehicle or 50 ng/ml BDNF during 10 min. Numbers indicate ratio of protein/α-tubulin relative to time zero.

**Sup Fig. 8** **a.** Invasion of mo-shNC and mo-shTrkB 4t1BR5 cells in organotypic brain slices using sholl analysis. *Left*: distribution of the median # of intersections away from initial sphere edge 48h after seeding. *Right:* Total # of new intersections. **b** Full WB images showing pro-BDNF and BDNF in 4T1BR5 cells treated with vehicle, 10nM E2, 50ng/ml BDNF, CM-OH or CM-E2 in figure 5e, using two different BDNF antibodies. Left: SC H-117 (SC20981) , Right: ab ERP1292 (Ab108319).

**Sup Fig. 9** a. Single channel images of GFAP, BDNF and DAPI corresponding to merged colored-image shown in Fig. 5f.

**Sup Fig 10. a** Time-course of BDNF signaling in 231BR cells treated with BDNF. **b** Signaling pathways activated in serum-starved 231BR cells treated for 30 min with vehicle, 10 nM E2, 50 ng/ml BDNF, CM-OH or CM-E2. **c.** TrkB mRNA levels in 231Br cells stably expressing two different TrkB- targeting shRNAs or a non-targeting control (PLK)1).
