# Supplementary figures and images for: Estradiol induces BDNF/TrkB signaling in triple-negative breast cancer to promote brain metastases
Source: Oncogene. 2019 Feb 22;38(24):4685–99. doi: 10.1038/s41388-019-0756-z (PMC6565485; doi:10.1038/s41388-019-0756-z)

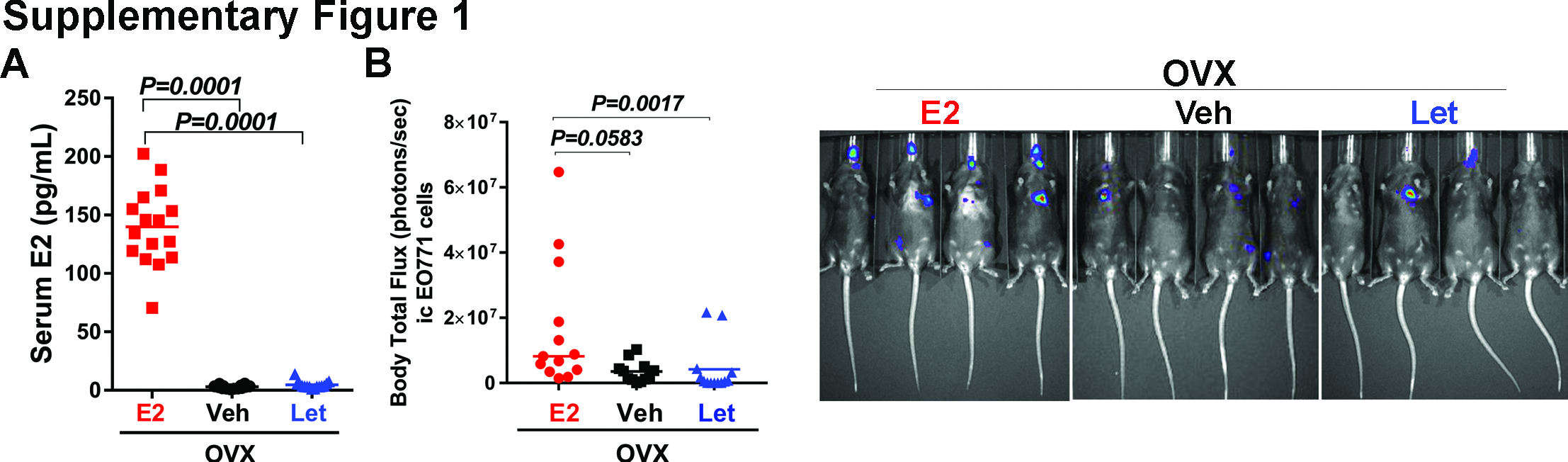

Supplement: Supplementary file 3 — Supplementary Figure 1. [file 41388_2019_756_MOESM3_ESM.tif]

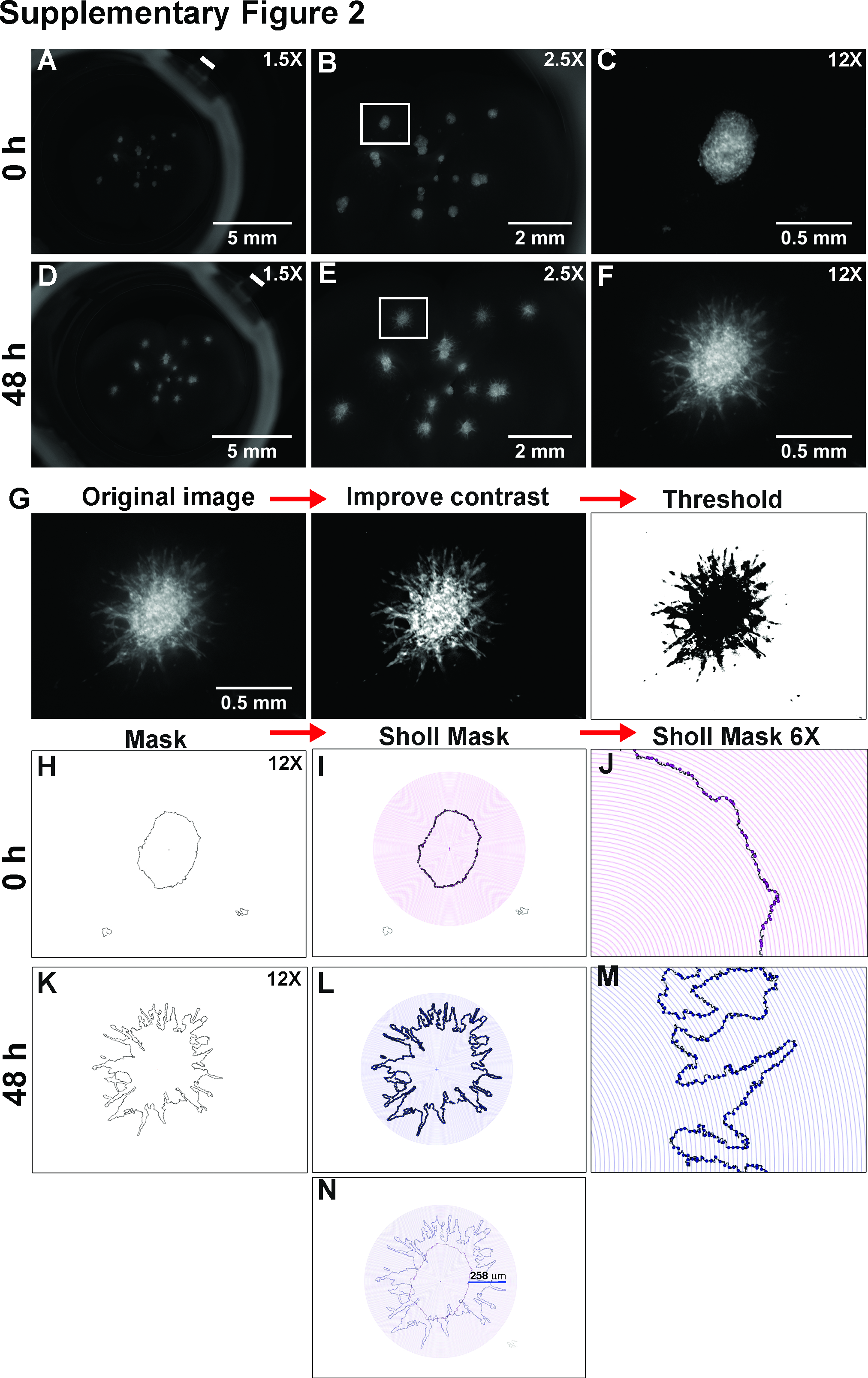

Supplement: Supplementary file 4 — Supplementary Figure 2. [file 41388_2019_756_MOESM4_ESM.tif]

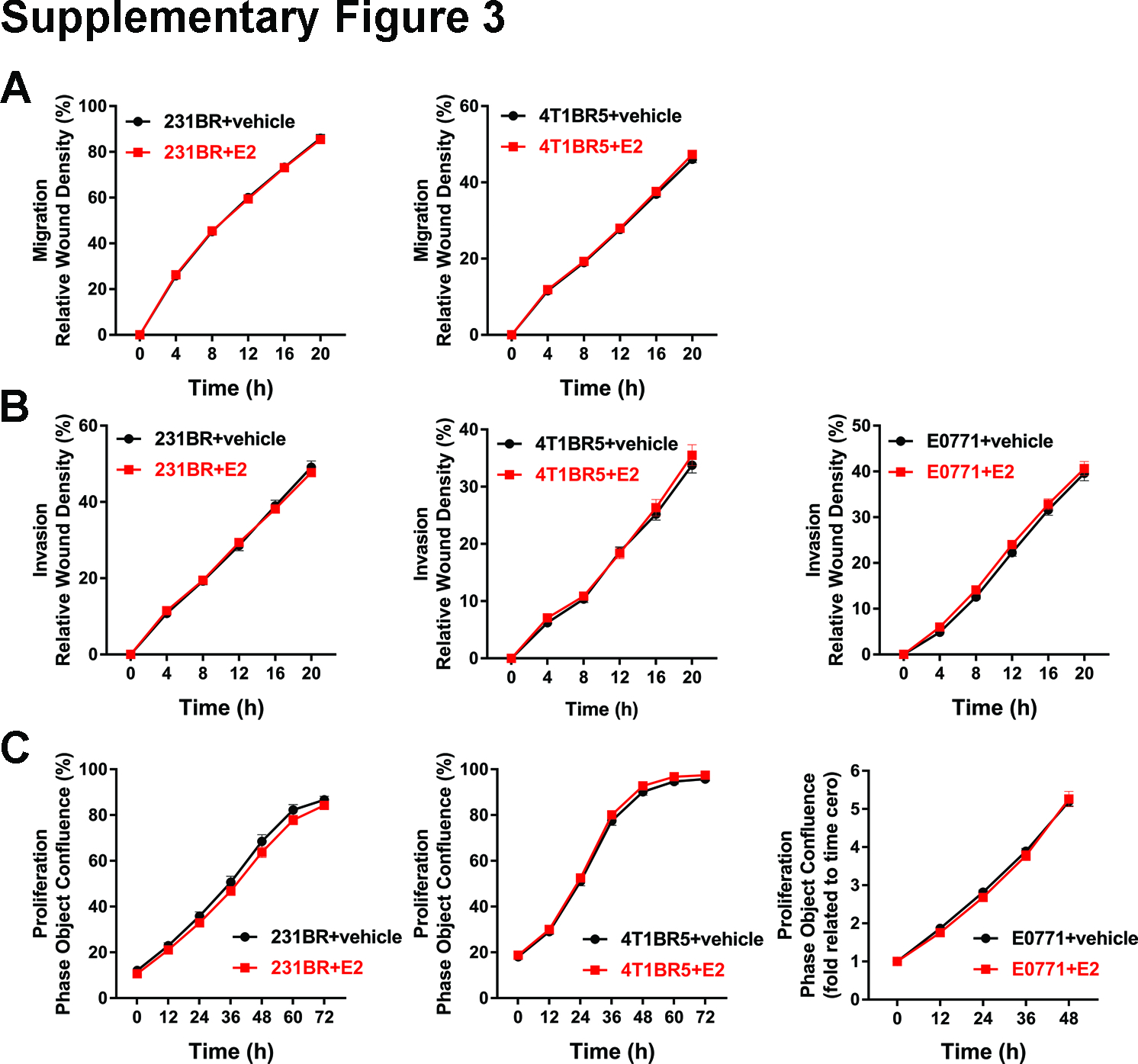

Supplement: Supplementary file 5 — Supplementary Figure 3. [file 41388_2019_756_MOESM5_ESM.tif]

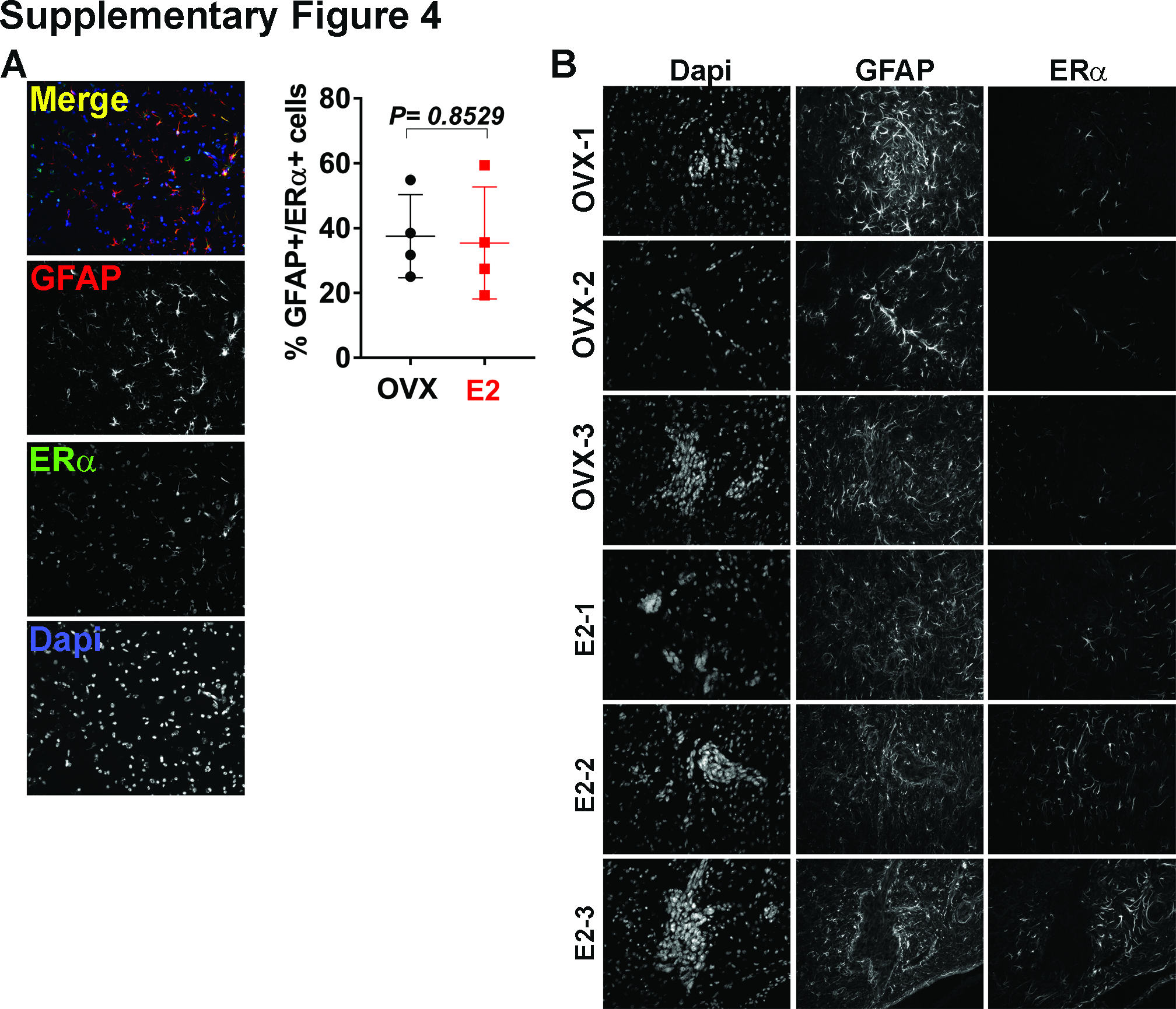

Supplement: Supplementary file 6 — Supplementary Figure 4. [file 41388_2019_756_MOESM6_ESM.tif]

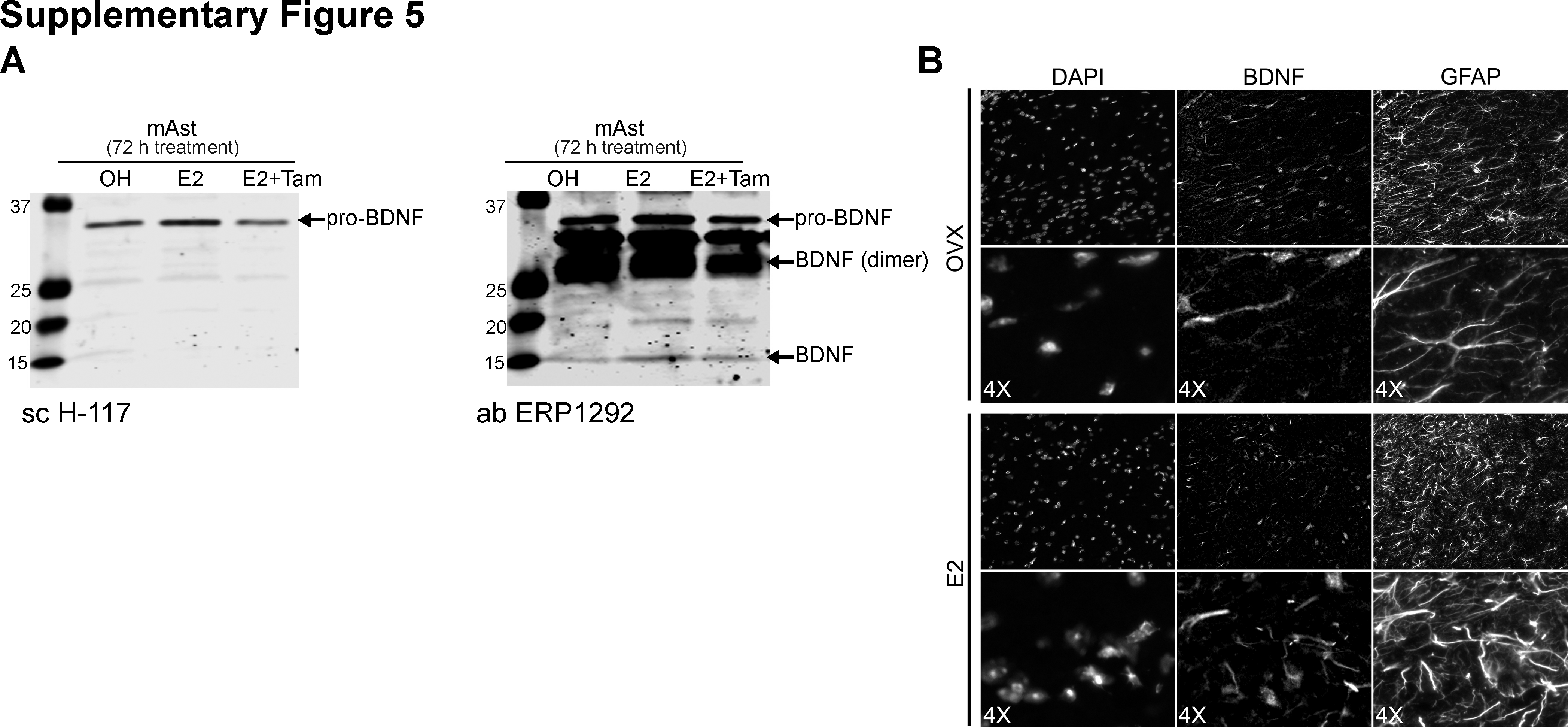

Supplement: Supplementary file 7 — Supplementary Figure 5. [file 41388_2019_756_MOESM7_ESM.tif]

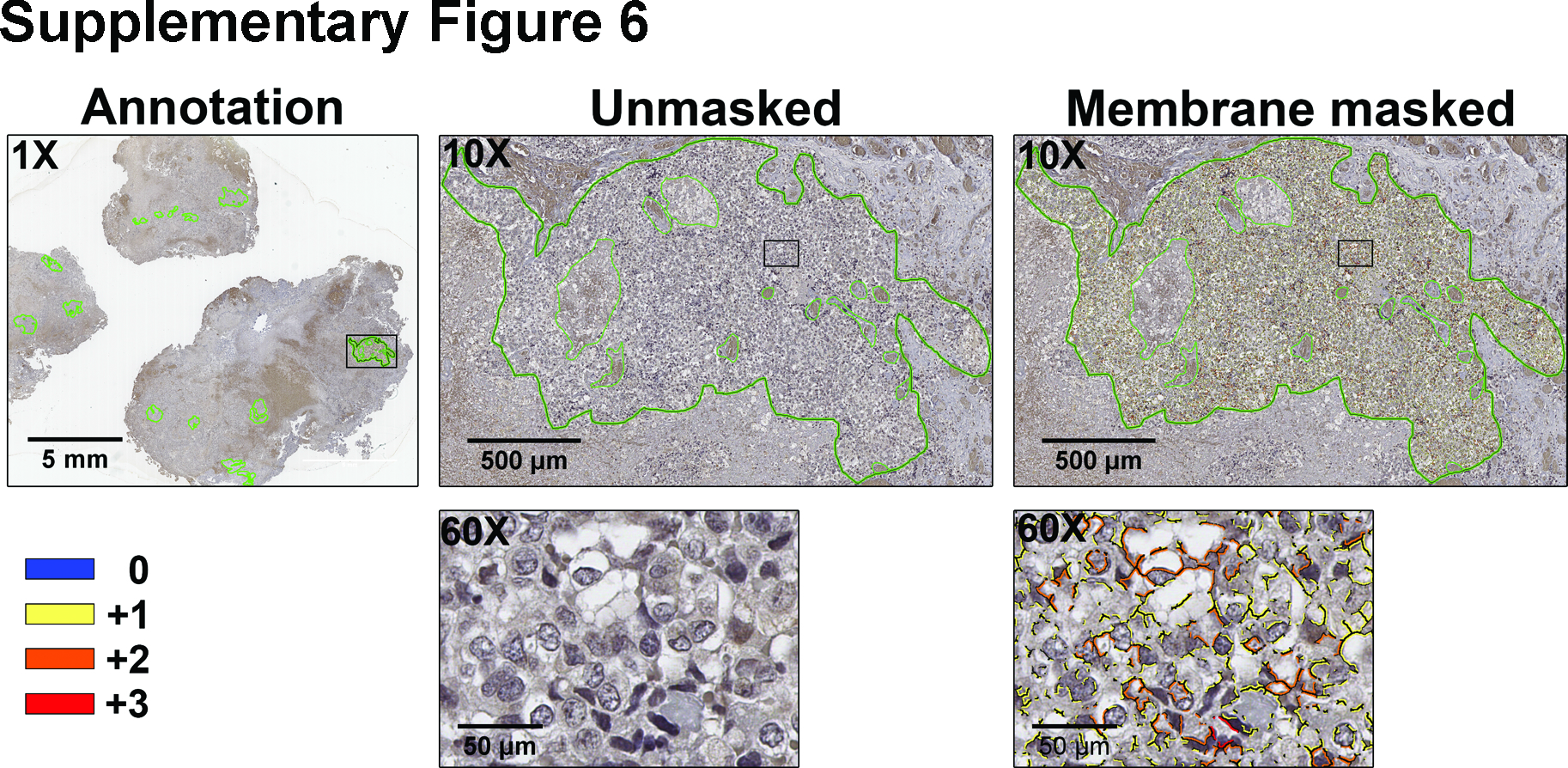

Supplement: Supplementary file 8 — Supplementary Figure 6. [file 41388_2019_756_MOESM8_ESM.tif]

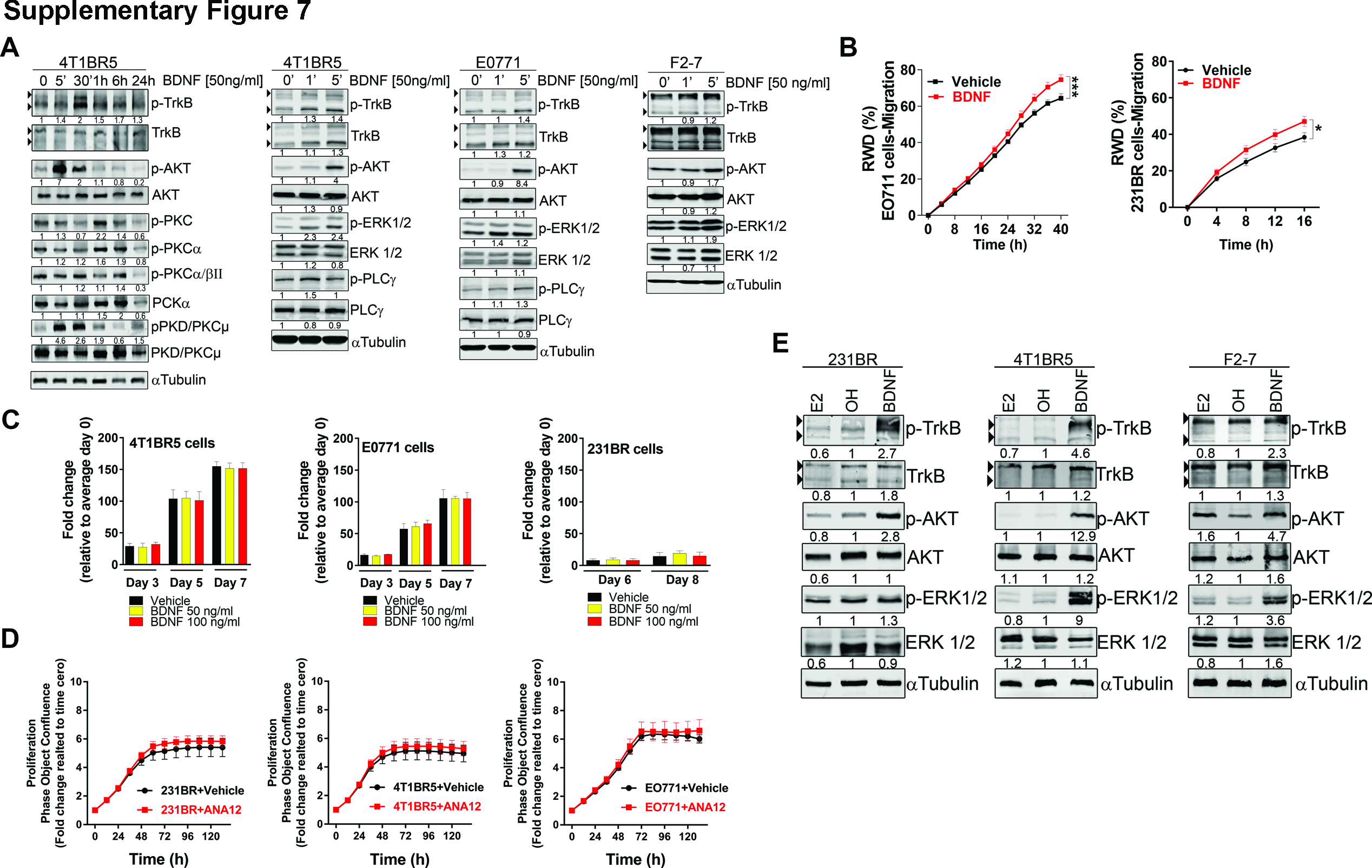

Supplement: Supplementary file 9 — Supplementary Figure 7. [file 41388_2019_756_MOESM9_ESM.tif]

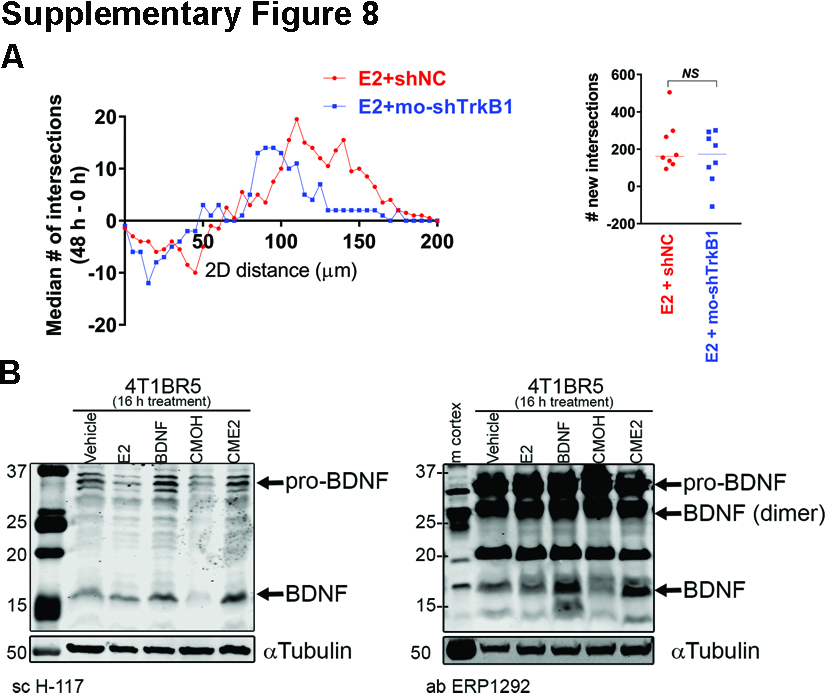

Supplement: Supplementary file 10 — Supplementary Figure 8. [file 41388_2019_756_MOESM10_ESM.tif]

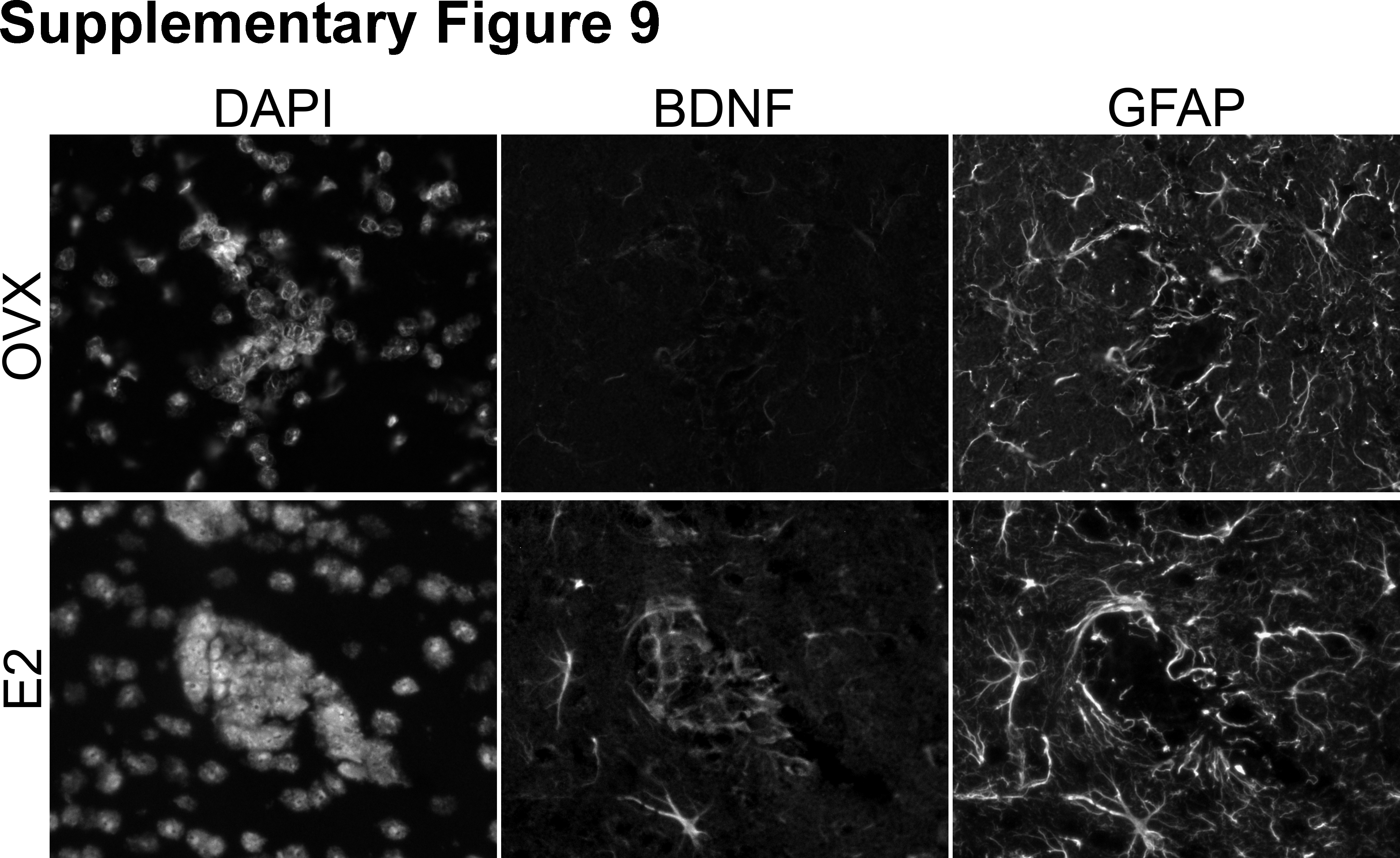

Supplement: Supplementary file 11 — Supplementary Figure 9. [file 41388_2019_756_MOESM11_ESM.tif]

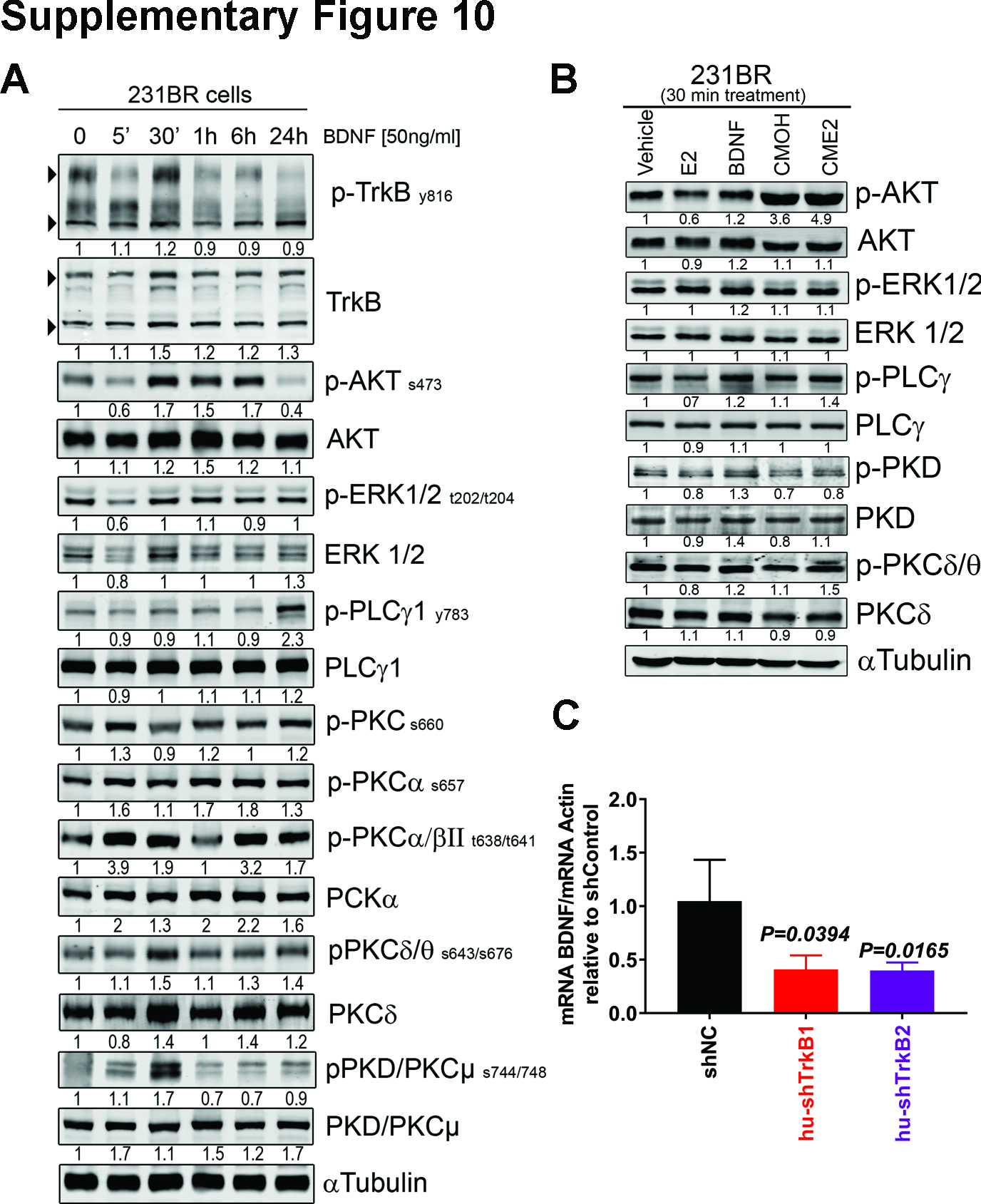

Supplement: Supplementary file 12 — Supplementary Figure 10. [file 41388_2019_756_MOESM12_ESM.tif]
